# Supplementary material for: Quantifying synergy and redundancy between networks
Source: Cell Rep Phys Sci. 2024 Apr 17;5(4):101892. doi: 10.1016/j.xcrp.2024.101892 (PMC11077508; doi:10.1016/j.xcrp.2024.101892)
Supplement: Document S1. Figures S1–S4 and Tables S1–S5 [file mmc1.pdf]

Cell Reports Physical Science, Volume *5*

Supplemental information

Quantifying synergy and redundancy between networks

Andrea I. Luppi, Eckehard Olbrich, Conor Finn, Laura E. Suárez, Fernando E. Rosas, Pedro A.M. Mediano, and Jürgen Jost

Supplemental Items

Supplemental Experimental Procedures

Alternative possible formulation of network redundancy for shortest paths

Throughout the paper, we have seen that the PND framework applied to global efficiency can quantify redundant, unique or synergistic contributions from path lengths alone. As natural as this seems, there are certain cases where this choice might clash with our intuitions for what each of these contributions should be. As an example, consider the shortest path between nodes 1 and 5 in the following two scenarios:

1. In network A_1 the shortest path is $1-2-3-5$. In network B_1 the shortest path is $1-2-3-4-5$. In $A_1 \cup B_1$ the shortest path is $1-2-3-5$.

2. In network A_2 the shortest path is $1-2-6-5$. In network B_2 the shortest path is $1-2-3-4-5$. In $A_2 \cup B_2$ the shortest path is $1-2-6-5$.

In both cases there is a unique contribution from A_1 with a path of length 4, so our current formulation considers these two cases as equivalent. Intuitively, however, some might argue that there is a sense in which A_2 is "more unique" (with respect to B_2) than A_1, because in A_2 the existence of edges $2-6$ and $6-5$ is unique and is what enables the shortest path, while in A_1 only the existence of $3-5$ is unique.

As another example where intuitions may differ, consider the shortest path between nodes 1 and 4 in the following two scenarios:

1. Network A_1 has edges $2-3-4$, but not $1-2$, and network B_1 has edges $1-2-3$, but not $3-4$. Suppose that in both networks there are other paths from 1 to 4 of length 5. Network $A_1 \cup B_1$ has a path $1-2-3-4$ which is of length 4.

2. Network A_2 has edges $3-4$, but not $1-2-3$, and network B_2 has edges $1-2-3$, but not $3-4$. Again, suppose that in both networks there are other paths from 1 to 4 of length 5. Network $A_2 \cup B_2$ has a path $1-2-3-4$ which is of length 4.

In this case, some might argue that A_2 and B_2 are "more synergistic" than A_1 and B_1 are, because in the second scenario, $2-3$ is common to both networks. But this is not reflected in our approach based on the efficiency gain provided by $A \cup B$ because in both cases you have $l_{A \cup B} = 4$ and $l_A = l_B = 5$.

Note however that these examples do not disagree that there is unique path from node 1 to node 5 in network A (for the first example), nor that there is synergy between networks A and B in the second example. The disagreement is not about the presence of uniqueness, or synergy, but only about how to quantify their extent. In other words, in terms of the three-question summary of our approach ("(1) Is there synergy? (2) If not, is there uniqueness? (3) If there is synergy or uniqueness, how much of it is there?") these examples agree on the answer to questions (1) and (2), and the divergence of intuitions only applies for question (3).

A natural alternative to the operationalisation of path redundancy that may address this concern would be one whereby shortest paths in networks A and B are redundant if and only if they involve traversing exactly the same edges (note that the two criteria coincide, for every shortest path, when network B is equal to network A). This "redundancy as identity" means that the same path would literally be available on both networks. However, we can easily see that this alternative definition of path redundancy suffers from a number of drawbacks. Mathematically, this option would be ill-defined in cases where multiple shortest paths of equal length exist, but do not coincide, with no natural way of "breaking ties". From a practical standpoint, identifying the length of the shortest paths between two nodes in networks A and B, and checking whether they are the same, is much simpler than (and a subset of) enumerating all possible shortest paths and determining whether any of them match. Finally, at a more conceptual level, in many real-world settings (e.g., travellers looking for synergies between two transport networks in going between two specific locations) the ease of reaching a destination node (in terms of time, money, or number of steps that can be saved) seems to be of far greater importance than the specific identity of the intermediate steps, or their contribution to these savings, for most purposes.

For these reasons, while we agree that there can certainly be cases where the identity and contribution of the specific sub-paths are of interest, we also believe that PND as presented is the more appealing and widely applicable alternative for the question of path efficiency (which we emphasise is only one possible application of PND) Nevertheless,we do not discard future variations of PND that take these considerations into account.

Supplemental proofs

This section contains supporting proofs for the properties of the efficiency-based Partial Network Decomposition (PND), presented in the Materials and Methods section of the main text. Our main goal is to prove that the proposed network redundancy function in Eq. (**??**) provides a non-negative PND of the network's average efficiency, such that $F_\partial^\alpha \geq 0, \forall \alpha$. We will do so by closely following the proofs for PID in Appendix D of Williams and Beer[1], bearing in mind the differences between our network redundancy function $F_\cap^\alpha$ and their information redundancy function $I_{\min}(S;\alpha)$. Note that some proofs in Ref.[1] do not depend on the properties of $I_{\min}(S;\alpha)$, and rely only on the structure of the antichain lattice —

which is shared between PID and PND. For further detail, please see the insightful discussion surrounding the non-negativity of certain PID measures in Appendix C of Ref.[2].

We begin by formally defining the efficiency f of a pair of distinct nodes $\omega = (v_1, v_2) \in \mathcal{V} \times \mathcal{V}$ s.t. $v_1 \neq v_2$ as the minimum length of all paths between them in the set of edges $\mathcal{E}$:

$$f(\omega; \mathcal{E}) = \min_{\mathtt{p} \in \mathtt{P}(\omega;\mathcal{E})} |\mathtt{p}|^{-1} \ , \tag{S1}$$

where $\mathtt{P}(\omega; \mathcal{E})$ is the set of all paths between v_1 and v_2 in $\mathcal{E}$. For convenience, we repeat the definition of the redundancy function presented in the main text,

$$f_\cap^\alpha(\omega) = \min_{a \in \alpha} f(\omega; \mathcal{E}^a) \ . \tag{S2}$$

In the following, we may omit the dependence on ω for simplicity of notation. The following theorems (up to Theorem 6) hold for all ω.

Theorem 1. *$f(\omega; \mathcal{E})$ is non-negative.*

Proof. Follows from the fact that path lengths are non-zero positive integers. Following standard convention, the shortest path length between disconnected nodes is taken to be positive infinity. □

Lemma 1. *$f(\omega; \mathcal{E}^a)$ increases monotonically under subset inclusion.*

Proof. Consider $a, b \subseteq \{1, ..., N\}$, with $a \subset b$. Recall that, by definition, $\mathcal{E}^a = \bigcup_{i=1}^k \mathcal{E}_{n_i}$ for any $a = \{n_1, .., n_k\} \subseteq \{1, \ldots, N\}$. If $a \subset b$, then $\mathcal{E}^a \subseteq \mathcal{E}^b$. Since the set of paths $\mathtt{P}(\omega; \mathcal{E})$ grows with the inclusion of more edges in $\mathcal{E}$, the minimum of any function on $\mathtt{P}$ must decrease with said inclusion, and therefore its inverse must increase. Thus, if $a \subset b$ then $f(\omega; \mathcal{E}^a) \leq f(\omega; \mathcal{E}^b)$. □

Theorem 2. *$f_\cap^\alpha$ increases monotonically in the redundancy lattice.*

Proof. This proof depends only on the structure of the redundancy lattice and Lemma 1, both of which are shared between Ref.[1] and this work. Proof is exactly as in the original work, replacing $I_{\min}(S; \alpha)$ by $f_\cap^\alpha$ and $I(S = s; a)$ by $f(\omega; \mathcal{E}^a)$. □

Theorem 3. *f_∂^α can be written in closed form as*

$$f_\partial^\alpha = f_\cap^\alpha - \sum_{k=1}^{|\alpha^-|} (-1)^{k-1} \sum_{\substack{\mathcal{B} \subseteq \alpha^- \\ |\mathcal{B}| = k}} f_\cap^{\bigwedge \mathcal{B}} \ . \tag{S3}$$

Proof. This proof depends only on the structure of the redundancy lattice and Eq. (??) defining f_∂^α as the Moebius inversion of $f_\cap^\alpha$, both of which are shared between Ref.[1] and this work. Proof is exactly as in the original work, replacing $I_{\min}(S; \alpha)$ by $f_\cap^\alpha$ and $\Pi_{\mathbf{R}}(S; \alpha)$ by f_∂^α. □

Theorem 4. *f_∂^α can be written in closed form as*

$$f_\partial^\alpha = f_\cap^\alpha - \max_{\beta \in \alpha^-} \min_{b \in \beta} f(\omega; \mathcal{E}^b) \ . \tag{S4}$$

Proof. First we note that, for the redundancy lattice, the following statement holds:

$$\alpha \wedge \beta = \underline{\alpha \cup \beta} \ , \tag{S5}$$

where $\underline{X}$ is the set of minimal elements of a poset X (see Refs.[1,3] for a proof). Combining Eqs. (S2) and (S3) yield

$$f_\partial^\alpha = f_\cap^\alpha - \sum_{k=1}^{|\alpha^-|} (-1)^{k-1} \sum_{\substack{\mathcal{B} \subseteq \alpha^- \\ |\mathcal{B}| = k}} \min_{b \in \bigwedge \mathcal{B}} f(\omega; \mathcal{E}^b) \ , \tag{S6}$$

and by Lemma 1 and Eq.(S5),

$$= f_\cap^\alpha - \sum_{k=1}^{|\alpha^-|} (-1)^{k-1} \sum_{\substack{\mathcal{B} \subseteq \alpha^- \\ |\mathcal{B}| = k}} \min_{\beta \in \mathcal{B}} \min_{b \in \beta} f(\omega; \mathcal{E}^b) \ . \tag{S7}$$

Then, applying the maximum-minimums identity[1] we have

$$= f_\cap^\alpha - \max_{\beta \in \alpha^-} \min_{b \in \beta} f(\omega; \mathcal{E}^b) \ , \tag{S8}$$

Or, equivalently, by applying Eq. (S2) again,

$$= f_\cap^\alpha - \max_{\beta \in \alpha^-} f_\cap^\beta \ . \tag{S9}$$

□

Theorem 5. *f_∂^α is non-negative.*

Proof. If α is the infimum of $\mathcal{A}$, $f_\partial^\alpha = f_\cap^\alpha$, and $f_\partial^\alpha \geq 0$ follows from Theorem 5. If α is not the infimum of $\mathcal{A}$, $f_\partial^\alpha \geq 0$ follows directly from Theorems 2 and 4. □

Theorem 6. *F_∂^α is non-negative.*

Proof. Follows directly from Theorem 5 and the fact that $F_\partial^\alpha = \mathbb{E}\left[f_\partial^\alpha\right]$ is a weighted sum of non-negative quantities with non-negative weights. □

As noted by Chicharro and Panzeri[2], the crucial aspect of this proof (in addition to f being non-negative and monotonically increasing) is that $f_\cap^\alpha$ is defined as a minimum of a set of values, with each value associated with a set of sources. Although the overall PND framework is applicable to other utility functions f and other redundancy functions $f_\cap^\alpha$, these may not lead to a non-negative decomposition.

Supplemental figures and tables

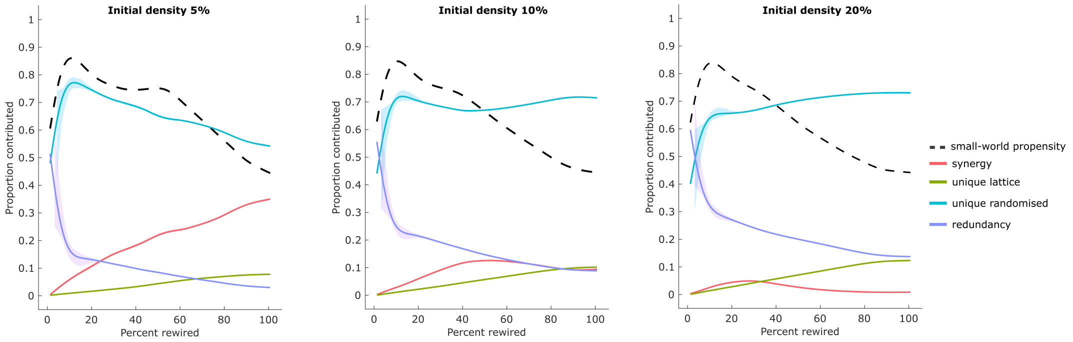

Figure S1. **Consistent effects of progressive rewiring, for networks of different density.** Synergistic, unique, and redundant contributions to global efficiency, as well as the network small-world propensity, are shown as a function of the percentage of rewired edges, for 5%, 10%, and 20% density.

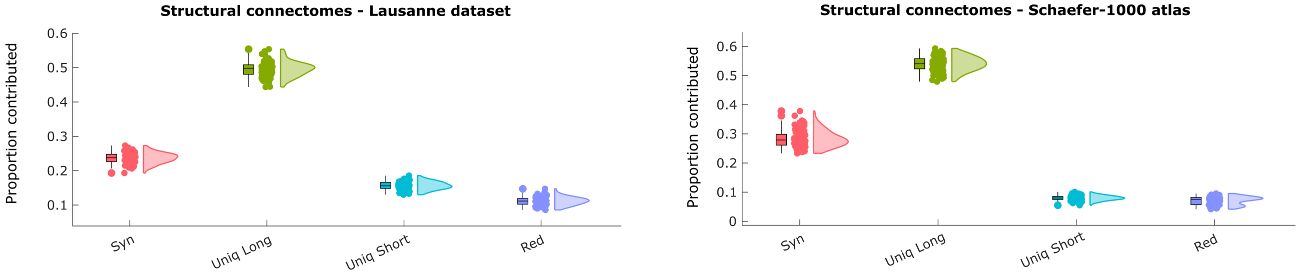

Figure S2. **Replication with alternative reconstruction of the human structural connectome**. Left: connectomes reconstructed from an independent dataset of Diffusion Spectrum Imaging data (N=70 subjects), using the Lausanne anatomical parcellation with 234 cortical and subcortical regions. Right: connectome reconstructed from an alternative sub-parcellation of the Schaefer functional atlas with 1000 cortical nodes (N=100 subjects). Y-axis: proportion of shortest paths accounted for by each PID term. Box-plots indicate the median and inter-quartile range of the distribution. Each data-point is one subject.

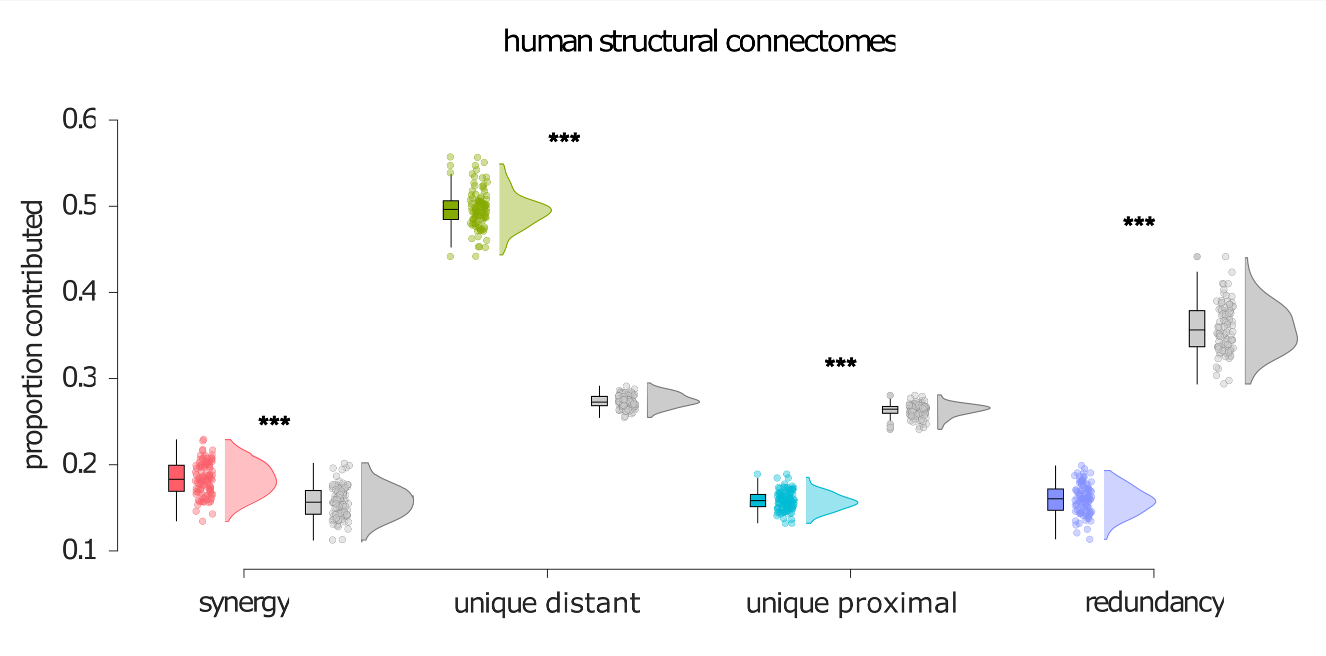

Figure S3. **Comparison against degree-preserving randomised networks**. Grey distributions indicate the corresponding values for degree-preserving randomised networks. ***: $p < 0.001$ against null distribution of values obtained from rewired null networks. Y-axis: proportion of shortest paths accounted for by each PND term. Box-plots indicate the median and inter-quartile range of the distribution. Each data-point is one subject (N=100).

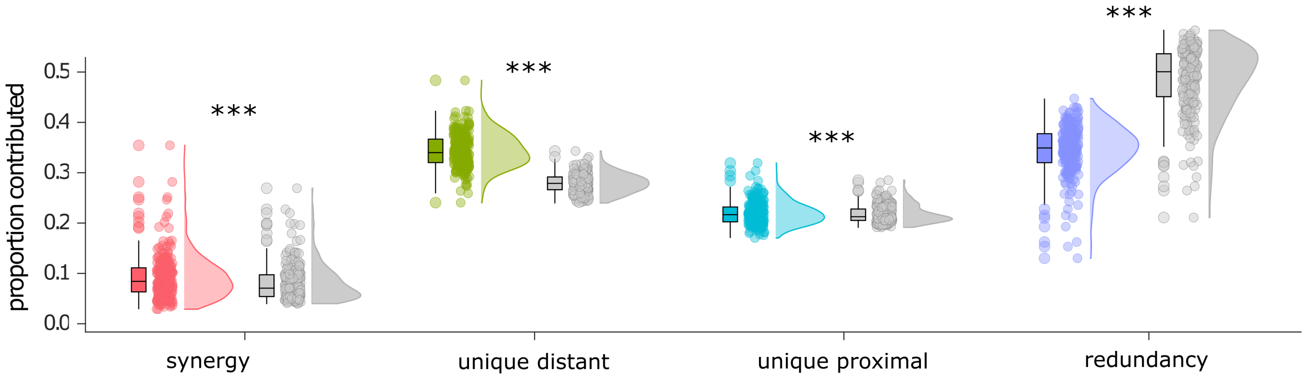

Figure S4. **Prevalence of synergistic, unique, and redundant efficiency contributions, comparing mammalian structural connectomes against degree-preserving randomised nulls.** Grey distributions indicate the corresponding values for degree- preserving randomised networks. ***: $p < 0.001$ against null distribution of values obtained from rewired null networks. Y-axis: proportion of shortest paths accounted for by each PND term. Box-plots indicate the median and inter-quartile range of the distribution. Each data-point is one animal (N=220, colored dots), or its corresponding null network (grey dots).

Contrast	Empirical Mean	Empirical SD	Null Mean	Null SD	tStat	df	pVal	Hedges g	95% CI Lower	95%CI Upper
**Synergy**	1.84e-01	1.94e-02	1.77e-01	2.08e-02	5.04	99	p <0.001	0.34	0.21	0.50
**Unique distant**	4.97e-01	2.15e-02	5.06e-01	1.16e-02	-5.53	99	p <0.001	-0.50	-0.72	-0.31
**Unique proximal**	1.58e-01	1.10e-02	1.59e-01	6.14e-03	-1.04	99	0.303	-0.10	-0.31	0.10
**Redundancy**	1.61e-01	1.72e-02	1.58e-01	2.03e-02	3.05	99	0.003	0.14	0.05	0.24

TABLE S1. Results for the statistical comparison between human structural connectivity networks, and corresponding geometry-preserving rewired nulls.

Contrast	Empirical Mean	Empirical SD	Null Mean	Null SD	tStat	df	pVal	Hedges g	95% CI Lower	95%CI Upper
**Synergy**	1.84e-01	1.94e-02	1.33e-01	2.02e-02	33.89	99	p <0.001	2.56	2.29	2.91
**Unique distant**	4.97e-01	2.15e-02	2.60e-01	7.27e-03	111.42	99	p <0.001	14.68	13.03	17.14
**Unique proximal**	1.58e-01	1.10e-02	2.50e-01	8.11e-03	-59.16	99	p <0.001	-9.46	-10.65	-8.57
**Redundancy**	1.61e-01	1.72e-02	3.56e-01	2.99e-02	-116.18	99	p <0.001	-7.98	-9.08	-7.20

TABLE S2. Results for the statistical comparison between human structural connectivity networks, and corresponding degree-preserving rewired nulls.

Contrast	Empirical Mean	Empirical SD	Null Mean	Null SD	tStat	df	pVal	Hedges g	95% CI Lower	95%CI Upper
**Synergy**	2.29e-01	3.45e-02	1.64e-01	2.31e-02	22.93	99	p <0.001	2.22	1.97	2.57
**Unique distant**	2.71e-01	4.95e-02	3.71e-01	3.94e-02	-33.07	99	p <0.001	-2.21	-2.65	-1.91
**Unique proximal**	2.67e-01	3.40e-02	1.91e-01	1.39e-02	24.56	99	p <0.001	2.92	2.59	3.38
**Redundancy**	1.83e-01	3.74e-02	2.31e-01	3.47e-02	-20.42	99	p <0.001	-1.33	-1.57	-1.15

TABLE S3. Results for the statistical comparison between human functional connectivity networks, and corresponding geometry-preserving rewired nulls.

Contrast	Empirical FC Mean	FC SD SD	SC Mean	SC SD	tStat	df	pVal	Hedges g	95% CI Lower	95%CI Upper
**Synergy**	2.29e-01	3.45e-02	1.84e-01	1.94e-02	14.23	99	p <0.001	1.62	1.40	1.91
**Unique distant**	2.71e-01	4.95e-02	4.97e-01	2.15e-02	-39.13	99	p <0.001	-5.88	-6.85	-5.23
**Unique proximal**	2.67e-01	3.40e-02	1.58e-01	1.10e-02	29.96	99	p <0.001	4.27	3.80	4.94
**Redundancy**	1.83e-01	3.74e-02	1.61e-01	1.72e-02	6.94	99	p <0.001	0.75	0.52	1.04

TABLE S4. Results for the statistical comparison between human structural connectivity networks, and corresponding functional connectivity networks, thresholded to have the same network density.

Contrast	Empirical Mean	Empirical SD	Null Mean	Null SD	tStat	df	pVal	Hedges g	95% CI Lower	95%CI Upper
**Synergy**	9.19e-02	4.46e-02	6.07e-02	4.22e-02	48.52	219	p <0.001	0.72	0.61	0.88
**Unique distant**	3.43e-01	3.38e-02	3.61e-01	4.24e-02	-9.66	219	p <0.001	-0.45	-0.55	-0.35
**Unique proximal**	2.19e-01	2.31e-02	1.81e-01	1.54e-02	31.22	219	p <0.001	1.96	1.77	2.20
**Redundancy**	3.45e-01	4.94e-02	3.98e-01	5.93e-02	-32.95	219	p <0.001	-0.96	-1.12	-0.83

TABLE S5. Results for the statistical comparison between mammalian structural connectivity networks, and corresponding geometry-preserving rewired nulls.

References

[1] Paul L Williams and Randall D Beer. Nonnegative decomposition of multivariate information. *arXiv:1004.2515*, 2010.

[2] Daniel Chicharro and Stefano Panzeri. Synergy and redundancy in dual decompositions of mutual information gain and information loss. *Entropy*, 19(2):71, 2017.

[3] Jason Crampton and George Loizou. Two partial orders on the set of antichains. *Research note*, 2000.
