# Supplementary material for: Quantifying synergy and redundancy between networks
Source: Cell Rep Phys Sci. 2024 Apr 17;5(4):101892. doi: 10.1016/j.xcrp.2024.101892 (PMC11077508; doi:10.1016/j.xcrp.2024.101892)
Supplement: Document S2. Article plus supplemental information [file mmc3.pdf]

Cell Reports
Physical Science

CellPress
OPEN ACCESS

Article

Quantifying synergy and redundancy between networks

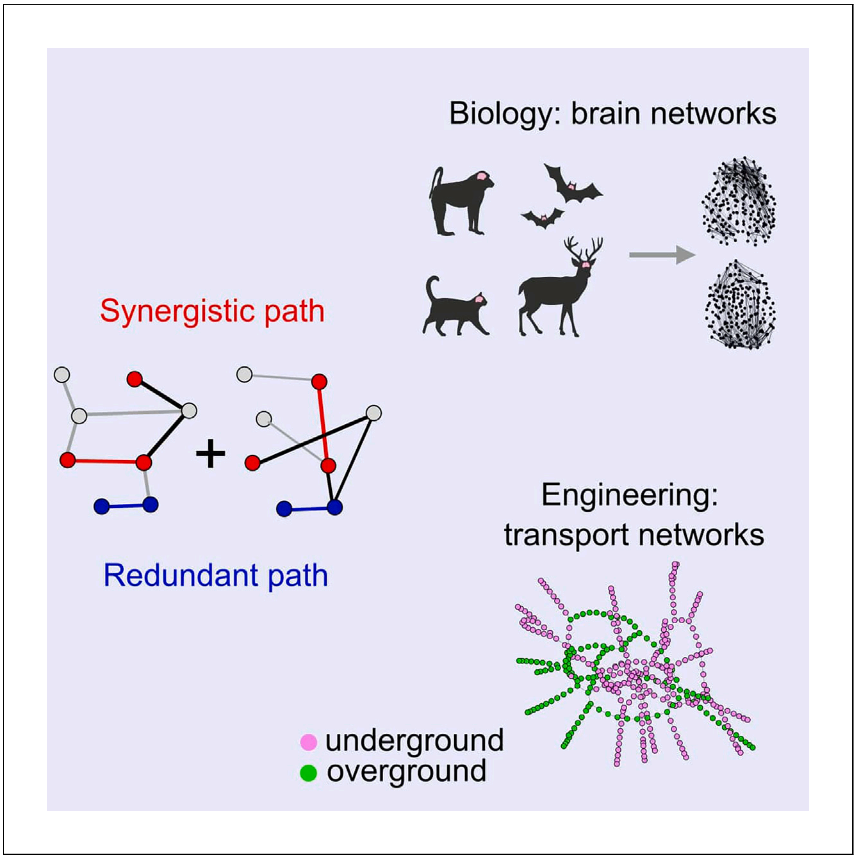

Andrea I. Luppi, Eckehard Olbrich, Conor Finn, Laura E. Suárez, Fernando E. Rosas, Pedro A.M. Mediano, Jürgen Jost

al857@cam.ac.uk (A.I.L.)
f.rosas@sussex.ac.uk (F.E.R.)
pmediano@imperial.ac.uk (P.A.M.M.)

Highlights

We quantify how complementary, redundant, or distinct two networks are

This framework is applicable from transport networks to brain networks of 124 species

Short paths in mammalian brains rely primarily on long-range connections

Synergy between long-range and short-range connections is conserved across species

Networks provide a powerful way of understanding complex systems. When are two networks redundant or complementary? Synergy can facilitate efficiency, but redundancy provides robustness. The framework presented by Luppi et al. could help decide how to trade off different desiderata when designing network systems or evaluate their presence in biological, social, or artificial networks.

Luppi et al., Cell Reports Physical Science 5, 101892
April 17, 2024 © 2024 The Authors.
https://doi.org/10.1016/j.xcrp.2024.101892

Cell Reports Physical Science

CellPress OPEN ACCESS

Article

Quantifying synergy and redundancy between networks

Andrea I. Luppi,[1,2,3,4,12,*] Eckehard Olbrich,[5] Conor Finn,[5] Laura E. Suárez,[4] Fernando E. Rosas,[3,6,7,8,*] Pedro A.M. Mediano,[9,*] and Jürgen Jost[5,10,11]

SUMMARY

Understanding how different networks relate to each other is key for understanding complex systems. We introduce an intuitive yet powerful framework to disentangle different ways in which networks can be (dis)similar and complementary to each other. We decompose the shortest paths between nodes as uniquely contributed by one source network, or redundantly by either, or synergistically by both together. Our approach considers the networks' full topology, providing insights at multiple levels of resolution: from global statistics to individual paths. Our framework is widely applicable across scientific domains, from public transport to brain networks. In humans and 124 other species, we demonstrate the prevalence of unique contributions by long-range white-matter fibers in structural brain networks. Across species, efficient communication also relies on significantly greater synergy between long-range and short-range fibers than expected by chance. Our framework could find applications for designing network systems or evaluating existing ones.

INTRODUCTION

Networks provide an intuitive representation of systems comprising interacting components, enabling the use of rigorous mathematical tools to study complex systems across domains of science. A useful way of leveraging network representations involves assessing the (dis)similarity between the topologies of two networks. For example, one may want to compare the train and flight transportation networks linking a given set of cities, compare different modes of social interaction within the same group of people, or contrast different types of connections bridging different brain areas. Approaches to this question are usually based on comparing different network topologies in terms of their "distance." In effect, various scalar metrics to capture the divergence between different networks have been introduced for this purpose, including graph edit distance,[1] graph kernel methods,[2] spectral methods based on the graph's eigenspectrum,[3–5] and information-theoretic metrics based on various graph properties,[6,7] among others[8–14] (reviewed in Wills and Meyer,[15] Tantardini et al.,[16] and Ahmad et al.[17]). However, these approaches tend to characterize the divergence between networks in terms of a single number, thus yielding a one-dimensional (1D) description of a potentially rich discrepancy.

Here, we introduce a new framework that characterizes the similarity between networks using a multidimensional approach, which brings to light different ways in which networks can be (dis)similar and—most importantly—complementary to each other. Our approach draws inspiration from the literature on partial information

[1]Division of Anaesthesia and Department of Clinical Neurosciences, University of Cambridge, Cambridge, UK

[2]St John's College, University of Cambridge, Cambridge, UK

[3]Centre for Eudaimonia and Human Flourishing, University of Oxford, Oxford, UK

[4]Montreal Neurological Institute, McGill University, Montreal, QC, Canada

[5]Max Planck Institute for Mathematics in the Sciences, Leipzig, Germany

[6]Department of Informatics, University of Sussex, Brighton, UK

[7]Centre for Complexity Science, Imperial College London, London, UK

[8]Centre for Psychedelic Research, Department of Brain Sciences, Imperial College London, London, UK

[9]Department of Computing, Imperial College London, London, UK

[10]ScaDS.AI, Leipzig University, Leipzig, Germany

[11]Santa Fe Institute, Santa Fe, NM, USA

[12]Lead contact

*Correspondence: al857@cam.ac.uk (A.I.L.), f.rosas@sussex.ac.uk (F.E.R.), pmediano@imperial.ac.uk (P.A.M.M.)

https://doi.org/10.1016/j.xcrp.2024.101892

Check for updates

This is an open access article under the CC BY license (http://creativecommons.org/licenses/by/4.0/).

decomposition (PID), which develops the principle that information is not a monolithic entity but can be disentangled into qualitatively different types, including redundancy (information that can be independently retrieved from more than one source), uniqueness (information that can be retrieved only from a specific source), and synergy (information that cannot be retrieved from a single source but can only be retrieved by taking into account multiple sources at once).[18–21] Inspired by the PID framework, in this paper we introduce "partial network decomposition" (PND): a formalism that disentangles the relationship between networks in terms of different "similarity modes," quantifying the extent to which two or more networks are redundant, unique, or synergistic. To illustrate the broad applicability of this framework, we showcase how PND provides new insights into real-world artificial and biological networks by analyzing two scenarios: London's public transportation systems and the topologies of short- vs. long-range connections in the brains of humans and 124 other mammalian species.

RESULTS

Conceptualizing synergy and redundancy between networks

Our central question is whether two networks can be considered synergistic or redundant or whether they provide unique contributions. Intuitively, two networks can be said to be redundant if the combination of the two is in some sense equivalent to each one of them separately. Conversely, one could say that they are synergistic if, when considered together, the two networks complement each other in some sense. It is important to emphasize that PND is not a replacement, but an extension, of existing techniques based on network similarity. Although redundancy may be understood as a measure of similarity by itself, our framework provides a richer characterization that also incorporates qualitatively different ways in which networks can differ.

Although our mathematical framework is more general, here we showcase it with an example on communication via the most efficient paths between nodes (in terms of number of steps, or time, or monetary cost), which is relevant for many real and artificial networks. Let us illustrate this principle in the case of transport networks: when considering the topologies of, e.g., bus and train networks, synergy arguably occurs if the most efficient way to move between stations—in terms of lowest cost or time or fewest steps—involves using both modalities. In contrast, the two networks are redundant if each provides alternative routes of equal efficiency and their combination does not provide additional gains. In other words, for the specific application to path efficiency, our approach can be summarized by two questions: (1) Does the most efficient path between two nodes of interest involve both networks (i.e., is there synergy between them)? (2) If not, is the most efficient path equally short in both networks, or is one more efficient than the other (i.e., distinguishing between uniqueness and redundancy)? If desired, the specific value of the synergy or uniqueness can be further quantified by answering an additional question: (3) What is the gain in efficiency against the next-best alternative? (See the supplemental experimental procedures for further discussion.)

To capitalize on this intuition, let us consider two undirected binary networks defined on the same set of nodes, denoted by A and B (Figure 1). Note that this does not amount to requiring each node to have non-zero degree in each network: we require only that each node should have at least one connection in total (i.e., it should not be completely isolated). It is certainly possible—and common in practice—to have nodes that are part of only one network and not the other, such as stations that can be

CellPress
OPEN ACCESS

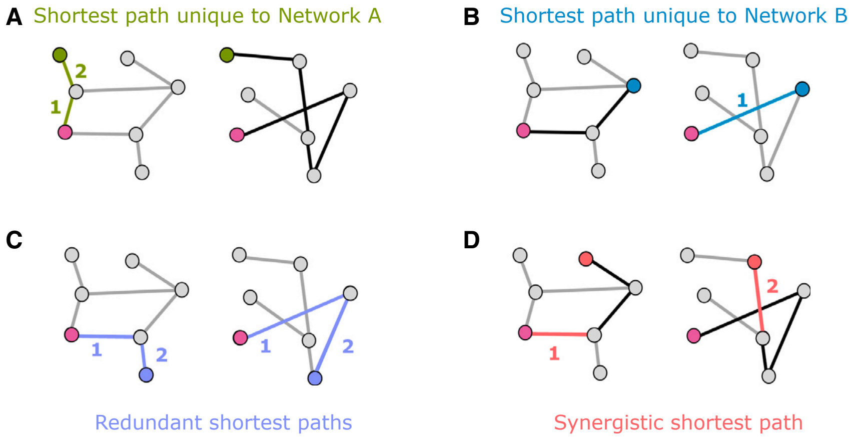

Figure 1. Illustration of the efficiency partial network decomposition between two networks
In each panel, the purple node is the starting point, and the other non-grey node is the destination. Green and light blue edges indicate shortest paths that are unique to each network (corresponding path in the other network is shown in black). Dark blue indicates redundant paths, which are equally long in either network; red indicates a synergistic path: shorter when the two networks are combined than in either individual network (shown in black).

reached by bus but not by train. In practice, any nodes that are part of only one network will be added as disconnected nodes on the other network (and vice versa). Simply, any such node would not be part of any redundant paths or any paths unique to the network where it does not have connections. In other words, the requirement is only that the union of the two networks should have no disconnected nodes. Nodes that are disconnected from both networks are simply ignored. We use binary (unweighted) networks to simplify our explanation, but our method also applies to weighted networks. For example, in the case of transport networks, some connections can be physically longer, or require more time, or be more expensive. Different applications may focus on different ways of assigning a weight to links, including weighted combinations of them.

Considering efficiency in terms of shortest path (fewest edges traversed), one can identify synergy between networks A and B whenever the most efficient (shortest) path between two nodes x and y involves traversing a combination of edges from A and B. (Although we frame our discussion largely in terms of shortest path lengths due to their more intuitive nature, our mathematical framework holds for any network measure of interest. For our numerical results, in particular, we use the efficiency [inverse of the shortest path] for its convenient mathematical properties, e.g., that it remains well defined [and equal to zero] for disconnected pairs.) Practically, this means that the most efficient path between x and y is found on the joint network, constructed by placing an edge between each pair of nodes that are directly connected in either network A or network B, such that the joint is also a binary undirected network. One can then operationalize efficient paths between nodes x and y over networks A and B as being redundant if they are of equal length—such that a traveler would be indifferent between traversing via network A or network B. Note that this differs from having multiple paths between x and y within network A or network B[22]: what we care about is that x and y be reachable in the same number of steps within network A and within network B. Finally, if the most efficient path between nodes x and y is shorter in network A (respective to B) than in network B (respective to A), then it is natural to label it as a "unique" contribution of network A (respective to B). Thus, if the most efficient path between nodes x and y is of length l_A when using edges

from only network A, of length l_B when using edges belonging to only network B, and of length $l_{A \cup B}$ when using edges from A and B, then the classification of the link between x and y can be done according to the following procedure:

(1) synergistic if $\min\{l_A, l_B\} > l_{A \cup B}$;
(2) unique if $\min\{l_A, l_B\} = l_{A \cup B}$ and also $\max\{l_A, l_B\} > l_{A \cup B}$;
(3) redundant if $\max\{l_A, l_B\} = l_{A \cup B}$.

It is direct to verify that this procedure identifies the most efficient (shortest) path between a pair of nodes x and y as being synergistic, redundant, or unique to either network A or network B, with no other outcome being possible. Also, note that, although $l_A = l_B$ (the condition whereby the shortest path is of the same length in both networks A and B) is a necessary condition for the path between A and B to be redundant, it is not sufficient: combining the two networks may result in a shortest path that is shorter than the shortest in either original network (i.e., synergy). Therefore we use the more elaborate condition $\max\{l_A, l_B\} = l_{A \cup B}$ for redundancy: while less straightforward, it correctly reflects that redundancy occurs only when combining the two networks does not lead to an improvement (which would be a case of synergy). In other words, our definition of redundancy applied to shortest-path communication is that the shortest paths in the two original networks are of the same length and that the length does not diminish in the combined networks.

Following this rationale, we can obtain a global quantification of the prevalence of synergistic, redundant, and unique paths across the two networks in terms of the proportion of all shortest paths that they respectively account for. This procedure answers the following question: how many of the possible maximally efficient paths between nodes would an agent know if they knew only network A, or only network B, or both?

In addition to a global-level assessment, our approach allows us to obtain further insight by focusing on the relevance of different scales (e.g., paths of different lengths). In effect, it is possible that the interactions between the two networks may be different for paths of different lengths—e.g., short paths may be more redundant, while longer ones are more synergistic. Our method provides a straightforward way to obtain such insight: since it provides a decomposition of the efficiency between each pair of nodes, one can simply group the network's shortest paths in terms of their length l and check the proportion of them that are synergistic, redundant, or unique (note that length-1 paths, which correspond to direct edges, by definition can only be either redundant or unique).

Finally, we emphasize that this running example of shortest paths in two networks is merely a special case of the more general PND introduced here. The full framework is applicable to any network measure of interest and any number of networks (potentially weighted, directed, or with non-identical nodes) and is described in detail in the experimental procedures.

Partial network decomposition in random network models

To build some initial intuitions on how different networks may contribute to the most efficient paths of a combined network, we constructed pairs of binary undirected Erdős-Rényi networks of different densities (ranging from 1% to 100%) and evaluated the synergistic, redundant, and unique contributions between them. Results show that shortest paths become less synergistic and more redundant as the density of links grows (Figure 2A). In effect, the majority of maximally efficient paths on the joint

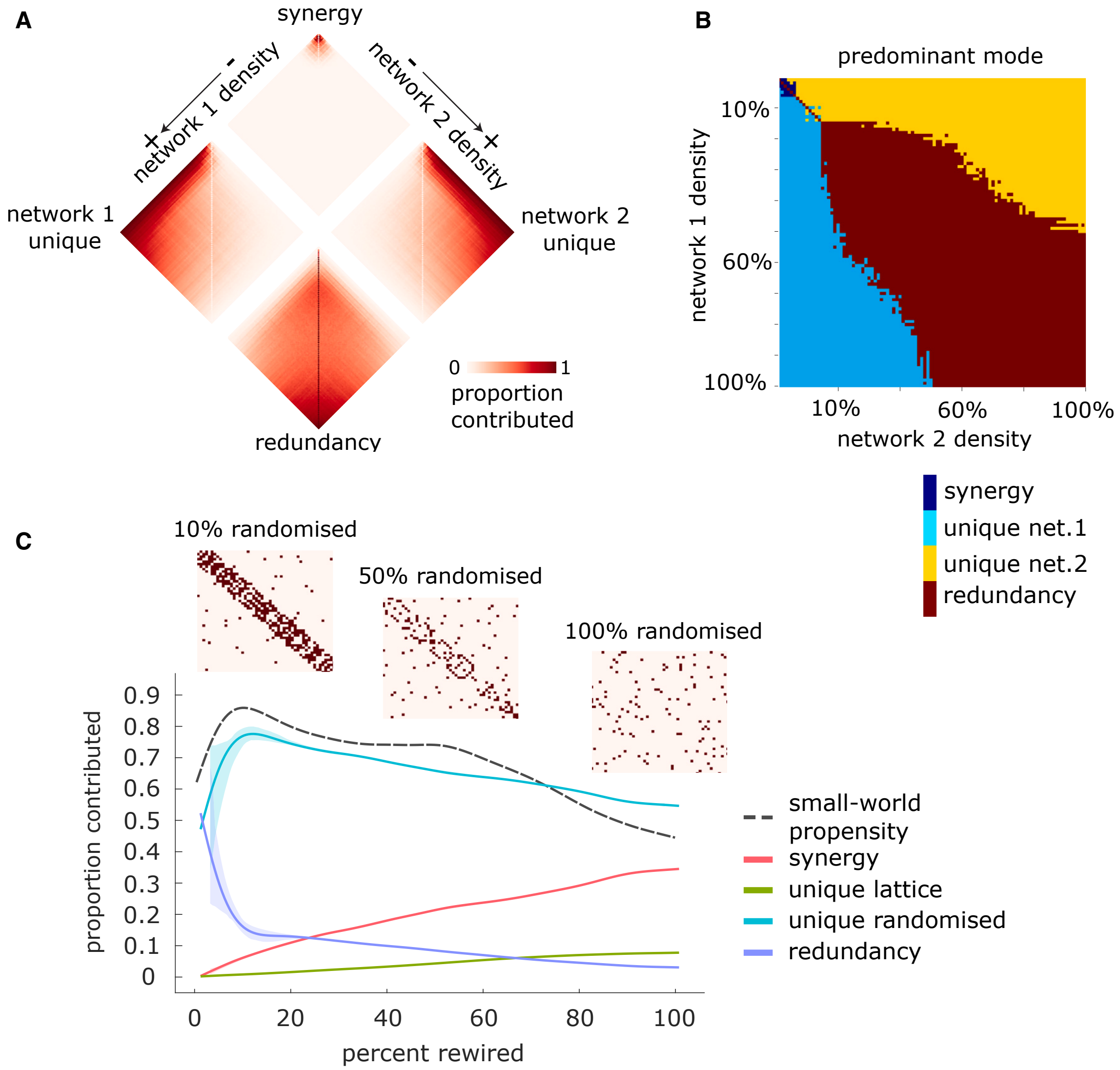

Figure 2. Contributions to shortest paths as a function of network density and rewiring

(A) Erdős-Rényi networks: each contribution is shown as a function of both networks' densities.

(B) Erdős-Rényi networks: component accounting for the majority of shortest paths, as a function of both networks' densities.

(C) Lattice and randomized networks: contributions are shown as a function of the percentage of rewired edges.

network are synergistic when the networks are both sparse (i.e., both with densities of 5% or less). Synergy is also present up to approximately 15% density and thereafter drops off rapidly. Unique contributions from one network tend to dominate when the other network is below approximately 15% density and thereafter also level off rapidly. Thus, when the two networks' densities are imbalanced, unique contributions from the denser one tend to predominate. Conversely, when the two networks have similar density (and both above 15% density), then the majority of the maximally efficient paths are redundant, with redundancy increasing gradually with both networks' density (Figure 2B).

To further develop intuitions, we also used PND to investigate the small-world character of networks.[23] Our setup considered two copies of the same lattice network and progressively randomly rewired one of them by 1% increments while preserving

the degree sequence. For each level of randomization, we calculated the efficiency PND between the original lattice network and its randomized counterpart (Figure 2C). Our results reveal two clearly distinct regimes. At low levels of randomization, redundancy dropped very rapidly as randomization increased, replaced by a prominent unique contribution from the rewired network and also an increasing prevalence of synergy between the two. Then, after a threshold of randomization had been surpassed (approximately 9% in Figure 2C), the unique contribution from the randomized network reached its peak and began to decline, while redundancy slowed its decline and began to plateau (Figure 2C). In contrast, both synergy and the unique information of the non-rewired lattice grew consistent with the degree of rewiring. Remarkably, we observed that the small-world propensity index[24] (see experimental procedures) of the joint network peaked at the same point of the unique information of the rewired network. Note that, since the joint network is obtained by combining the original lattice and its randomized counterpart, its density increases, as the randomization means that there are more and more non-overlapping edges. However, the small-world propensity is unaffected by density.[24] These results are largely unaffected by the density of the starting lattice network, replicating across 5%, 10%, and 20% density: an inflection point for both redundancy and the unique contribution of the randomized network is observed around 9% − 10% of rewiring, coinciding with the maximum value of small-world propensity (Figure S1). At higher network densities, the unique contribution of the randomized network can increase further after this inflection point with additional randomization, although not as steeply.

Taken together, these results show that PND can provide rich insights about the relationship between two networks. Building on these insights, in the following sections we show results of this machinery applied to real-world networks, both artificial and biological.

Low but significant redundancy of the London transport network

We analyzed real data from a context that may be familiar to everyday life—transportation networks. We investigated the topologies of two means of transportation in London: underground (i.e., subway) and overground (including different types of local trains) (Figure 3A). We used PND to gain insight on how these two types of transportation serve the needs of Londoners to move within the city. Note that not every station is served by both underground and overground: any node that has connections in only one network is included in the other network as a disconnected node.

Our analyses reveal that there is extremely low redundancy between the topologies of underground and overground. Other similarity modes, however, exhibit a strong dependence on the length of the path. For example, short paths show an approximately 70% unique contribution from underground and nearly 30% contribution from overground. Synergy rises rapidly with path length, accounting for over 60% of all paths at its peak and over 50% of paths of most lengths. This changes rapidly at the very longest paths, where again underground unique paths predominate, eventually reaching 100% contribution (Figure 3B). Overall, these findings speak about the efficiency of the design of these networks, which serve the city with almost no redundancy and substantial synergy.

To evaluate the significance of these findings, we compared the obtained results against the decomposition arising from a null distribution involving randomly rewiring both networks, while preserving the degree sequence to account for the

CellPress
OPEN ACCESS

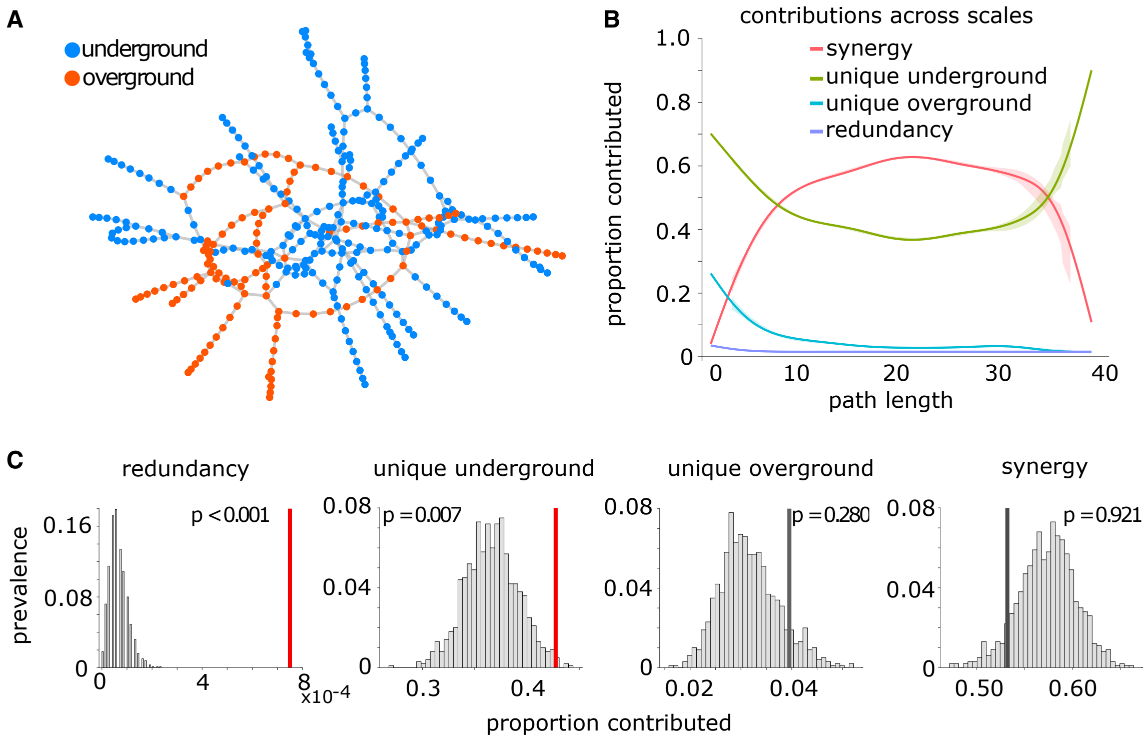

Figure 3. London transport networks

(A) The underground network (blue) and the overground network (orange).

(B) The relative proportion of shortest paths accounted for by redundant, unique, and synergistic contributions, as a function of path length.

(C) Empirical results (vertical lines) against a null distribution (gray histograms) obtained from degree-preserving randomization of the two networks. Red line indicates statistical significance ($p < 0.05$).

potential confounding effects of this low-level network property (Figure 3C). We found that the London transport network relies on unique contributions from the underground network significantly more than would be expected by chance ($p = 0.007$). Intriguingly, although redundancy is by far the least prevalent term in the decomposition, its value is nevertheless significantly greater ($p < 0.001$) than what would be expected based on two random networks of equal density and degree sequence (Figure 3C). The other two contributions (overground unique and synergy) did not significantly differ from their degree-preserving null counterparts. However, when null distributions were obtained using purely random networks (with the same density as the original ones) instead of degree-preserving random networks, the unique contribution of the overground network was also significantly greater than expected from chance ($p < 0.001$), whereas the contribution of synergy was significantly lower than expected from chance ($p = 0.002$). Thus, we see that the degree sequence plays a role in determining the decomposition.

Synergy of connectivity networks in the human brain

The next step in our analysis was to investigate the relationship between the networks of white-matter fibers that link spatially proximal regions (i.e., short-range fibers) and spatially distant regions (i.e., long-range fibers) within the human brain (note that we use “short-range/proximal” and “long-range/distal” to refer to the physical [Euclidean] distance between the two regions they connect—not to be confused with the shortest path between regions, which is in terms of the number of hops on the network). White-matter fibers provide the anatomical scaffold over

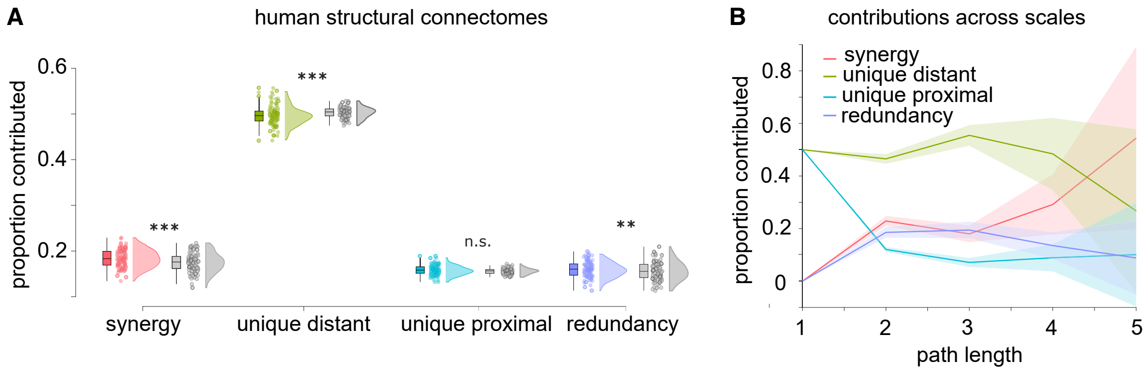

Figure 4. Prevalence of synergistic, unique, and redundant efficiency contributions for networks of white-matter fibers between spatially proximal and spatially distant regions of the human brain

(A) Proportion of the most efficient paths accounted for by each PND term. Boxplots indicate the median and interquartile range of the distribution. Each data point is one subject (n = 100). Gray distributions indicate the corresponding values for degree- and geometry-preserving randomized networks. ***$p < 0.001$ against null distribution of values obtained from rewired null networks.

(B) Prevalence of synergistic, unique, and redundant contributions as a function of path length for human structural brain networks.

which communication unfolds in the brain; understanding how their networked organization supports brain function is a major research topic in neuroscience.[25]

We used the PND framework to investigate the relationship between the efficiency of networks of white-matter tracts connecting spatially proximal and spatially distant regions of the human brain, reconstructed from *in vivo* diffusion MRI tractography in 100 healthy human adults (see experimental procedures). For each subject, we defined one network as comprising the 50% of fibers connecting the most spatially distant regions in that subject's brain, in terms of greatest Euclidean distance, and a second network as comprising the 50% of white-matter fibers connecting the most spatially proximal regions (smallest Euclidean distance between them). Thus, the two networks have equal density. As a first (subject-level) analysis, we decomposed the similarity of connections between spatially proximal and spatially distant regions observed in each subject.

Results revealed that, on average across individuals, nearly 50% of the maximally efficient paths in the overall structural connectome (combining all white-matter tracts) are accounted for by long-range connections between spatially distant regions, while synergistic paths are the second-largest contributors (Figure 4A). This suggests that long-range fibers play a key role in enabling shortest-path communication between regions that are distant, not only physically, but also topologically (the most efficient path between them involves many hops), despite being more metabolically expensive. In addition to this high-level description, however, our approach can also provide more detailed information. We began by considering paths of different lengths: our framework revealed that synergistic contributions become prevalent for paths comprising multiple hops (Figure 4B). This may be expected, since the longer the path, the more occasions there may be for making it more efficient with an appropriately placed connection.

To gain more insight on these results, we repeated our decomposition on consensus networks obtained using a procedure to aggregate individual connectomes,[26] which provides two networks that are representative of the topology of long-range and short-range white-matter fibers in the human brain (see experimental procedures). By decomposing the similarity of this representative pair of networks, we see again

CellPress
OPEN ACCESS

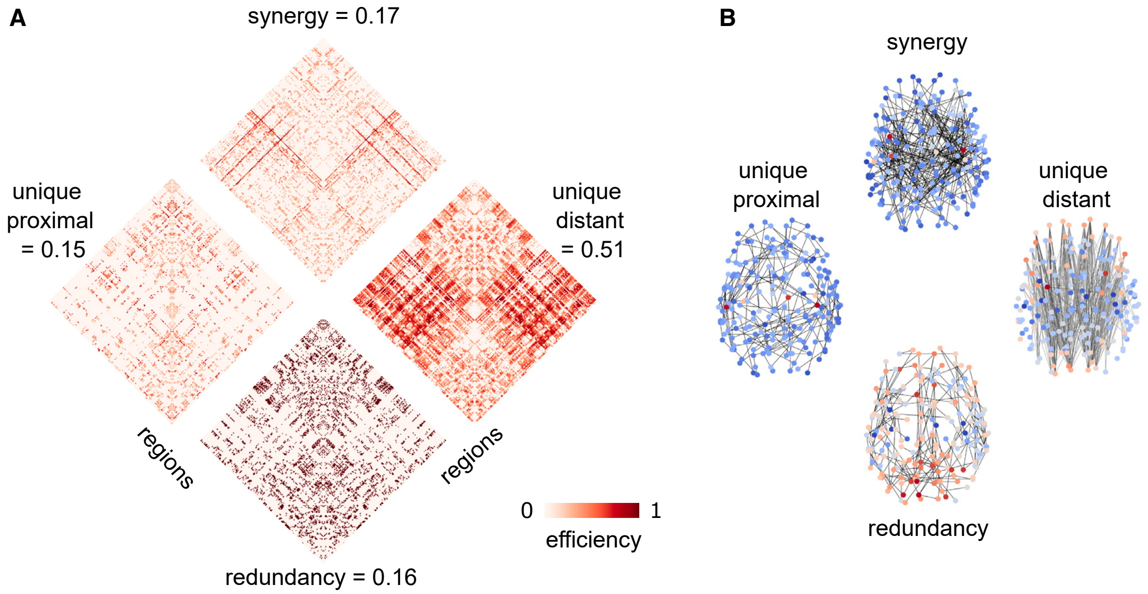

Figure 5. Edge-wise decomposition into synergistic, unique, and redundant efficiency contributions for group-consensus networks of white-matter fibers between spatially proximal and spatially distant regions of the human brain

(A) Each edge is assigned to the network of the corresponding mode, so that each edge is non-zero in only one of the four networks, and its value reflects the gain in path against the next best alternative. For each matrix, upper and lower quadrants correspond to interhemispheric connections, and right and left quadrants are interhemispheric.

(B) Same as (A), but with edges plotted in the brain to highlight distinct patterns of connectivity in the human brain. Warmer color indicates higher node degree. Networks are thresholded for visualization purposes.

that approximately 50% of the maximally efficient paths in the network are accounted for by white-matter tracts between spatially distant regions, confirming the results on individual subjects discussed above (Figure 5A). Importantly, these analyses allow us to study these networks at the level of individual edges. Results show that regions best reached via paths along long-range fibers are predominantly located in different hemispheres and at opposite ends of the anterior-posterior axis (Figure 5B). Cross-hemisphere connections are also prominent for synergistic paths. In contrast, redundant and uniquely short-distance paths are primarily located within the same hemisphere (Figure 5B). This is to be expected, since connecting physically distant nodes by traversing short-distance fibers inevitably involves many hops, making this a suboptimal strategy in terms of minimizing path length.

To demonstrate the robustness of our results, we show that the same pattern, with white-matter tracts between spatially distant regions accounting for most of the maximally efficient paths, can be replicated in an independent dataset of human diffusion MRI, which used diffusion spectrum imaging (Figure S2) and defined brain regions using an alternative anatomical parcellation, including subcortical structures. Likewise, we replicate the human results with a higher-resolution version of the Schaefer cortical parcellation (1,000 nodes); in this case, we see an even stronger contribution of synergy—although still second to the unique contribution of long-range tracts (Figure S2).

We then investigated the role of network topologies in shaping their respective contributions. For this, we considered a "geometry-preserving" rewired null model, preserving the degree sequence (and therefore density) of the original network, but also the approximate length of connections.[27] Examining rewired networks enabled us to

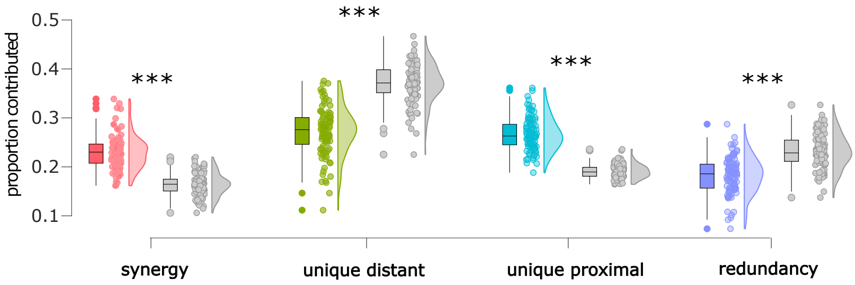

Figure 6. Prevalence of synergistic, unique, and redundant efficiency contributions for networks of functional connectivity between spatially proximal and spatially distant regions of the human brain

Gray distributions indicate the corresponding values for degree- and geometry-preserving randomized networks. ***$p < 0.001$ against the null distribution of values obtained from rewired null networks. The y axis presents the proportion of shortest paths accounted for by each PND term. Boxplots indicate the median and interquartile range of the distribution. Each data point is one subject (n = 100).

assess whether the results for the human structural connectomes could be observed just by chance for any random network with the same density, degree distribution, and spatial embedding. We see that if the networks are randomly rewired (while still preserving the degree sequence and spatial embedding of the original networks), synergy is significantly reduced, and redundancy is, too—although not as much (Figure 4A, gray plots). In contrast, the predominance of long-range connections observed in the empirical structural connectomes is further enhanced in geometry-preserving rewired nulls. Statistical comparisons confirm that the human connectome relies on both synergy and redundancy between long- and short-range tracts, significantly more than a geometry-preserving random network would. Instead, the geometry-preserving rewired networks rely on long-distance connections even more than empirical structural connectomes (Table S1). Notably, a different picture emerges when the null models are relaxed to still preserve the degree sequence, but not the length distribution (spatial embedding). In this case, synergy becomes the lowest contributor, and the highest contribution is not the unique contribution of long-distance connections anymore, but rather the redundancy (Table S2 and Figure S3).

Next, we considered empirical brain networks obtained from a different neuroimaging modality: correlation of functional MRI blood-oxygen-level-dependent (BOLD) signals (i.e., "'functional" connectomes). Unlike diffusion MRI, functional connectivity (FC) reveals connections between regions in terms of the similarity of their activity over time, thereby providing a different perspective on the network organization of the human brain. This enables us to ask whether any empirical brain networks will display the same pattern of results reported above, or whether those results are specific to the *structural* connectome.

Again, we consider one network of (functional) connections between spatially proximal regions and one network of connections between spatially distant regions for each individual. We observe a similar pattern compared with structural connectomes (Figure 6), with long-distance connections providing the highest contribution. However, the unique contribution of long-distance connections is nearly equaled by short-range connections and synergy, being less than one-third rather than nearly half of all the most efficient paths. Indeed, comparing human structural and functional connectomes from the same individuals (and with matched density), the

structural ones have significantly more unique contributions from long-range connections and significantly smaller contributions from each other kind (Table S4). Notably, geometry-preserving rewiring of the functional connectomes induces a similar pattern compared with the structural connectomes, with a further increase in the predominance of unique contributions from long-range connections and a drop in synergy—but also an increase rather than a decrease in redundancy (Figure 6; Table S3).

Thus, structural connectomes are dominated by unique contributions from fibers connecting spatially distant regions, whereas functional connectomes of equivalent density enjoy more equitable contributions across uniquely distal, uniquely proximal, and synergistic paths.

Synergy of structural brain networks across mammalian species

To evaluate if the obtained results are distinctive of the human structural connectome or if this is also observed in other species, we performed analogous analyses over a wide spectrum of structural connectomes from diffusion MRI, covering N = 220 individual animals from 124 mammalian species[4,28] (see experimental procedures).

We performed the same analysis as for the human structural connectomes, using Euclidean distance to divide white-matter tracts into those linking spatially proximal regions and those linking spatially distant regions (each accounting for 50% of the total edges, thereby ensuring two equally dense networks). We then identified the synergistic, redundant, and unique contributions to the global efficiency of the joint network. More similar to the human structural than the functional connectome, our results show that a substantial proportion of the most efficient paths in the network are accounted for by white-matter tracts between spatially distant regions (Figure 7). Results also show that mammalian structural connectomes are significantly more synergistic and significantly less reliant on white-matter tracts between spatially distant regions than corresponding degree- and geometry-preserving randomized null models (see Figure 7; Table S5). Unlike the human case, however, we find that non-human mammals also involve a substantial contribution of redundancy—which is nevertheless significantly less than what would be observed in random networks with the same spatial embedding.

As with the human structural connectomes, if the null models preserve only the degree sequence, and not the distribution of Euclidean distances, then synergy becomes the lowest contributor and the highest contribution is not the unique contribution of long-distance connections any more, but rather the redundancy (Figure S4).

DISCUSSION

Drawing inspiration from the field of information decomposition, here we introduced a simple but powerful framework to quantify qualitatively different aspects of the similarities and differences between two networks in terms of a graph measure of interest. When applied to global efficiency, which is related to communication using shortest paths, our approach determines the contribution of each network to the shortest-path communication on the joint network, assessing whether such contributions are redundant, unique to each of the two initial networks, or synergistic—a novel metric of the complementarity between two networks such that combining them makes some of the shortest paths even shorter. By considering network

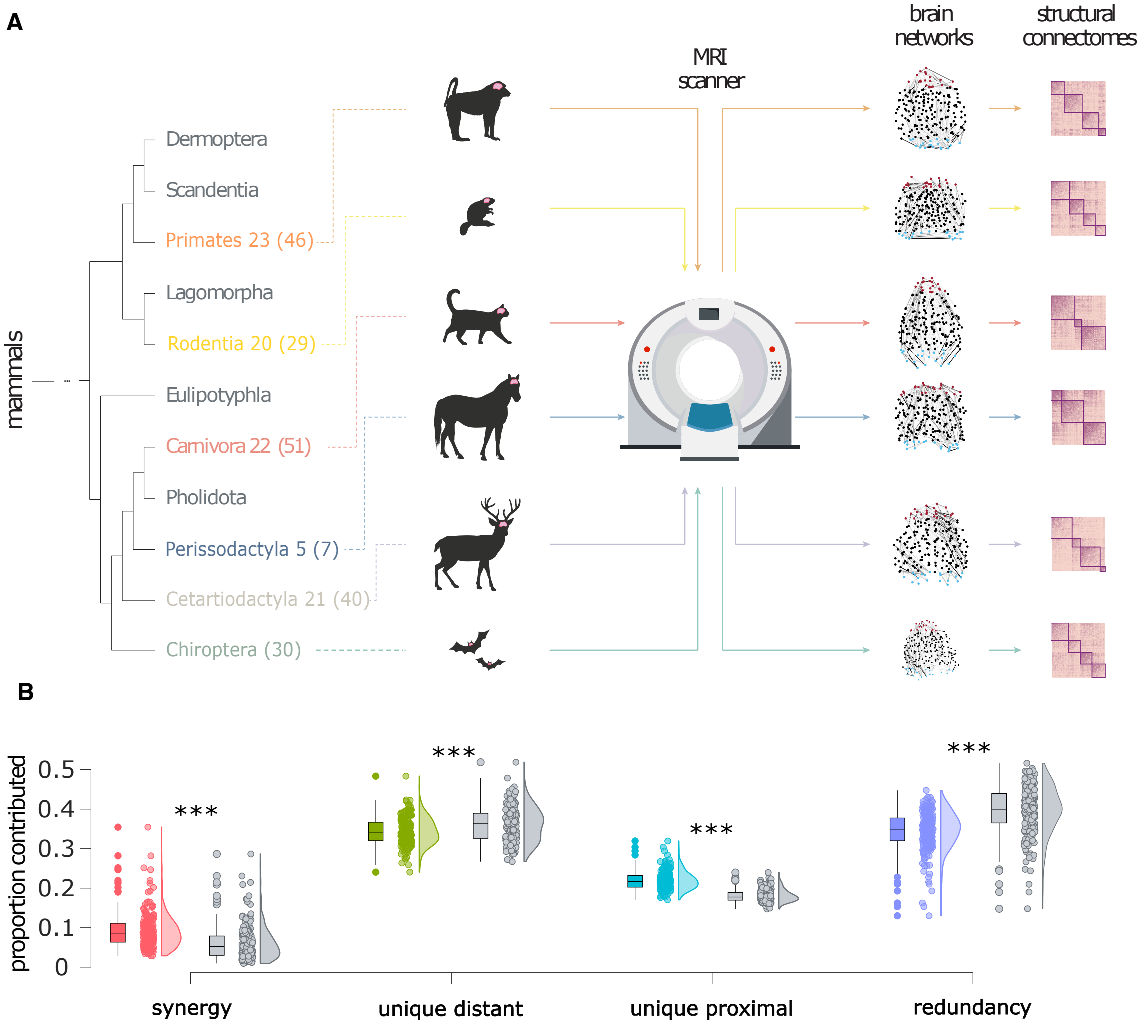

Figure 7. Prevalence of synergistic, unique, and redundant efficiency contributions for networks of white-matter fibers between spatially proximal and spatially distant regions of 220 mammalian brains

(A) The MaMI data set encompasses high-resolution *ex vivo* structural and diffusion MRI scans of 124 animal species; here, we show animals and corresponding brain networks representing the six taxonomic orders comprising the largest number of species (individual animals in parentheses), among those included here: Primates, Rodentia, Carnivora, Perissodactyla, Cetartiodactyla, and Chiroptera.

(B) Gray distributions indicate the corresponding values for degree- and geometry-preserving randomized networks. ***$p < 0.001$ against null distribution of values obtained from rewired null networks. The y axis represents the proportion of shortest paths accounted for by each PND term. Boxplots indicate the median and interquartile range of the distribution. Each data point is one animal (n = 220, colored dots) or its corresponding null network (gray dots).

efficiency, which is predicated on shortest paths, this approach takes into account the topology of the entire networks. It can also provide insights at different levels of resolution: from summary statistics (proportion of all shortest paths contributed by each mode), to information about their relative prevalence across paths of different length, to edge-level detail, including the number of steps saved with respect to the next-best alternative.

Our analyses illustrate how this approach can be applied to deepen our understanding of brain networks as well as transportation networks. In the case of London's transport networks, redundancy was found to be low in absolute terms but nevertheless significantly greater than that of random networks. In brain networks, PND

highlighted stark differences between the roles of long-range and proximal connections. We observed that reliance on long-range white-matter fibers for the majority of efficient paths in the network is a conserved feature of structural connectomes across mammalian species, including humans. This feature is not shared by just any brain network (it is not found in human functional connectomes) nor by random networks having the same degree distribution. On the contrary, we found that the brains of both humans and other mammals also exhibit significantly more synergy, and significantly less redundancy, than corresponding randomly rewired networks.

It is important to note that, unlike traffic networks, the brain does not rely solely (or perhaps even primarily) on communication via shortest paths, instead likely adopting a variety of mechanisms.[29–32] Therefore, our analysis of connectomes is not intended to quantify how signals actually propagate between brain regions—rather, our intention was to investigate what roles the topology of proximal and long-range connections in the brain would play in determining shortest paths.

In this sense, our analysis should be seen as analogous to the numerous investigations of brain small worldness and global efficiency,[24,28,33] which assess the network's suitability for shortest-path communication without claiming that such communication in fact happens. This approach is, therefore, distinct from studies that evaluate the roles of different communication strategies for explaining empirical patterns of interregional communication.[25,34]

In our study of random network models, the proposed framework showed that synergy generally predominates when both networks are very sparse (5% density or less; note that all our comparisons were performed against null models that preserved the original networks' density and degree, ensuring that such low-level properties do not explain our results). Conversely, synergy rapidly diminishes as the density of links in either network grows. This observation is noteworthy because a previous survey suggested that most biological and human-made networks are indeed sparse,[35] which, according to our findings, could be conducive to synergy.

Although it is apparent why synergy between networks can be advantageous, it is important to bear in mind that redundancy can also be valuable. The presence of redundant paths between nodes on two different networks means that transport will be unaffected, even if one of the two networks should fail. In contrast, failure of either network would easily jeopardize a synergistic path. In other words, redundancy of networks facilitates robustness, echoing previous findings in time-series analysis.[36]

Several future extensions of this framework are possible. First, we considered only unweighted, undirected networks. The PND method can be directly applied to weighted networks, provided that the weights can be combined across the two source networks—as would be the case, e.g., for prices or time, in the context of transport networks. Likewise, directed networks can also be straightforwardly accommodated. It is also worth acknowledging that transitioning between networks at a given node is not always possible and not always free of cost. This may need to be taken into account for future extensions. As an example, if the cost is time (for transport, for instance), then the time spent while waiting between different transport networks may need to be taken into account (e.g., airport layovers), whereas in terms of ticket price, there is often no monetary cost for switching between, e.g., different metro lines, but there can be a price for switching between metro and train. This would need to be taken into account when evaluating the advantage

conferred by synergy between the two networks. In particular, whenever there are exchange costs for transitioning between the two networks, by definition what will be affected is the synergy between them, which will be reduced. In other words, switching costs inherently reduce synergy—possibly to the point of eliminating it, at least for some paths. In the case of weighted networks, we expect that the extent to which switching costs affect synergy will depend on the specific weighting function for the edges, such as monetary cost or time.

Our approach requires a choice of how to operationalize the notion of "path redundancy." While in this work we have adopted equal length of shortest paths as an intuitive criterion for redundancy, this is not the only possibility. An interesting alternative is in terms of identity of edges traversed: will there be a path of length l between nodes x and y even if edge q is taken out? This may contribute to the characterization of redundancy as robustness. However, note that the present framework does not take into account whether multiple equally short paths between nodes x and y exist within network A. The existence of such redundant shortest paths within a network has also been proposed as a metric of robustness, although distinct from the one developed here.[37] Accounting for redundant shortest paths both within and between networks may provide a fruitful avenue for future extensions of the present work.

Also, the present framework relies on a measure of costs—here we used the global efficiency, which is based on shortest paths. However, different communication protocols can exist on networks: whereas shortest-path navigation requires knowledge of the global topology, other approaches can be agnostic, such as network diffusion based on random walks or navigability.[29–31] Incorporating different communication protocols over networks will be a valuable extension of our framework. One reason that shortest paths are especially appealing for our approach is that, when combining two networks (predicated on the same nodes, i.e., layers of a multiplex), shortest paths can never become longer: they only become shorter (if there is synergy between networks) or remain the same as the shortest of the two. Thus, the path efficiency can only increase or stay the same. However, this property is not guaranteed in the context of diffusion; unless the two networks are identical, the joint network will be denser than either of them, meaning that, on average, a random walker will have more options to choose between when leaving node x: although a shortcut to node y may now exist, the average number of steps to reach node y may still increase (because the random walker has more chances of choosing an edge that does not belong to the shortest path—and this repeats at every new node). Therefore, in its present form, our framework may often lead to no synergy being identified when considering communication via random walks.

Relatedly, here we did not consider the question of what dynamics take place over the network.[38,39] For instance, under dynamics that allow for the possibility of congestion (e.g., traffic), adding a shortcut may not always make travel on the network faster. On the contrary, it may even achieve the opposite effect by increasing congestion—a phenomenon known as "Braess's paradox."[40] Thus, two networks that are synergistic in terms of path length may nevertheless end up having "detrimental synergy" in terms of travel time, depending on the dynamics of navigation on the network. It will be a fruitful topic of future investigation to investigate such scenarios using our framework.

As a limitation, we note that our use of binary networks required us to threshold the FC (see experimental procedures), which imposes a somewhat arbitrary criterion

(although not devoid of ground[36]). However, this allowed us to keep the same edge density in the FC and structural connectivity networks, thereby removing the influence of density from our results. However, for the London transport example, the two networks had different densities of edges (underground vs. overground); this influences the prevalence of unique paths, since in a binary network an edge corresponds to the shortest (i.e., maximally efficient) possible path between the two nodes at its extremes, so a denser network will have more short paths (whether unique or redundant). Indeed, we found it to be so—but to an extent that was statistically unexpected based on density alone. Future work may extend the present framework to quantify the extent to which a network uses its available connections synergistically, uniquely, or redundantly, as a proportion of the theoretical maximum for a given partner network.

In conclusion, we have developed a simple yet versatile approach to characterize the relationship between two networks, taking into account topological properties and capable of providing both global and local insights. This work may find application when engineering network systems, to help decide how to trade off different desiderata (such as efficiency vs. robustness introduced by a new underground line) or evaluate their relative presence in existing systems, as we have done here for the human brain.

EXPERIMENTAL PROCEDURES

Resource availability

Lead contact

Further information and requests for resources and reagents should be directed to and will be fulfilled by the lead contact, Andrea Luppi (al857@cam.ac.uk).

Materials availability

This study did not generate new unique reagents.

Data and code availability

Data. The MAMI mammalian brain network data from Assaf et al.[28] are available at https://doi.org/10.5281/zenodo.7143143. Human Connectome Project (HCP) data in DSI Studio-compatible format are available at http://brain.labsolver.org/diffusion-mri-templates/hcp-842-hcp-1021. The network of London public rail transport is available from https://networks.skewed.de/net/london_transport.

Code. All original code is available in this paper's supplemental information, where we provide MATLAB code to compute the PND (Data S1).

Mathematical formalization

In this section we provide the mathematical foundations of our multiplex network analysis method. It is based on ideas from information theory, which we introduce below, and relies on a mapping between graphs and probability spaces. Beyond the specific metrics showcased in the results of this paper, this formalism paves the way for very general analyses of multiplex networks by combining principles of probability, graphs, and information theory.

Mapping probability theory and graphs

Let us consider a network defined by a pair $(\mathcal{V},\mathcal{E})$, where $\mathcal{V}$ is a set of vertices (or nodes) and $\mathcal{E}\subseteq\mathcal{V}\times\mathcal{V}$ is a set of (either binary or weighted) edges indexed by pairs of vertices. Our analyses focus on a "utility function," which corresponds to a network property of interest that depends on the set of edges $\mathcal{E}$. An example of such function

is the network's global efficiency, which is the average of the efficiency between each pair of nodes. For a given node pair $\omega = (v_1, v_2) \in \mathcal{V}\times\mathcal{V}$ in a network with edges $\mathcal{E}$, efficiency is defined as the inverse of the length of the shortest path between v_1 and v_2. In addition, we consider a probability measure $p(\omega)$ uniform over pairs of distinct nodes $\omega \in \mathcal{V}\times\mathcal{V}$, which establishes a random variable Ω. With this, the average value of a local utility function f over the network is given by:

$$F(\mathcal{E}) = \mathbb{E}[f(\Omega;\mathcal{E})], \qquad \text{(Equation 1)}$$

where $\mathbb{E}[f(\Omega;\mathcal{E})] = \sum_\omega f(\omega;\mathcal{E})p(\omega)$ is the expected value operator. In the example above, if $f(\omega,\mathcal{E})$ is the pairwise efficiency between two nodes, then F corresponds to the global efficiency of the whole network.

Now, suppose that we have two different sets of edges $\mathcal{E}_1$ and $\mathcal{E}_2$ for the same set of vertices $\mathcal{V}$, and we wish to compute how they affect the value of f. For example, a natural question is how much of the value of f can be attributed to the edges in $\mathcal{E}_2$, over and above those in $\mathcal{E}_1$. This can be naturally estimated as:

$$\Delta F(\mathcal{E}_1|\mathcal{E}_2) = F(\mathcal{E}_1 \cup \mathcal{E}_2) - F(\mathcal{E}_2). \qquad \text{(Equation 2)}$$

For example, if F is the global efficiency, then $\Delta F(\mathcal{E}_1|\mathcal{E}_2) \geq 0$ captures how much the efficiency increases by adding $\mathcal{E}_1$ to the links in $\mathcal{E}_2$.

Finally, note that the presented formalism can be extended to analyze networks with different sets of nodes by simply taking $\mathcal{V}$ to be the union of the sets of nodes in both networks. This will result in some nodes being disconnected in one of the networks, but as long as the utility function f remains well defined, so will the overall PND.

Simple example: Decomposing efficiency in two-layer networks

Using the established link between graphs and probability theory, one can take inspiration from frameworks to decompose information-theoretic quantities. In particular, here we use ideas from the PID framework[18] and develop a new set of tools to decompose the impact of various layers of edges on a given observable. Before presenting the general formalism, here, we illustrate the main ideas for a simple case of a two-layer network.

For this, let us consider a multilayer network $\mathcal{M}$ with vertices $\mathcal{V}$ and two sets of edges (i.e., layers) $\mathcal{E}_1, \mathcal{E}_2$. The full (or joint) network $\mathcal{E}$ contains all edges of both layers and mathematically is given by the union operator $\mathcal{E} = \mathcal{E}_1 \cup \mathcal{E}_2$. As an example, here, we decompose the global efficiency of the joint network $F(\mathcal{E})$ into qualitatively different types of contributions from $\mathcal{E}_1$ and $\mathcal{E}_2$.

Intuitively, for any given pair of vertices v_1, v_2 there are four possibilities: the efficiency could be equal in both layers of the network, it could be greater in one layer or in the other, or it could be greater in the joint network than in either layer. We refer to these different cases as "redundant," "unique," and "synergistic," respectively.

Let us now present a plausible (and empirically powerful) definition for each of these contributions. For a given pair of vertices v_1, v_2 we can take their redundant efficiency (or just redundancy) to be the minimum efficiency one could find by using either $\mathcal{E}_1$ or $\mathcal{E}_2$—that is, using the least efficient path fully contained within either of the layers. Mathematically, this can be written as:

$$r(v_1, v_2; \mathcal{M}) = \min_i f(v_1, v_2; \mathcal{E}_i). \qquad \text{(Equation 3)}$$

Based on this formula, the natural definition of the unique contribution of layer $\mathcal{E}_j$ is its gain in efficiency with respect to the redundancy for the same pair:

$$u_j(v_1, v_2; \mathcal{M}) = f\left(v_1, v_2; \mathcal{E}_j\right) - r(v_1, v_2; \mathcal{M}). \quad \text{(Equation 4)}$$

Note that, with this definition, for any given pair of nodes one layer will have zero unique contribution.

Finally, the synergistic contribution corresponds to the increase in efficiency seen in the joint network but not in either layer. Mathematically:

$$\begin{aligned} s(v_1, v_2; \mathcal{M}) &= f(v_1, v_2; \mathcal{E}) - (r(v_1, v_2; \mathcal{M}) \\ &+ u_1(v_1, v_2; \mathcal{M}) + u_2(v_1, v_2; \mathcal{M})) \\ &= f(v_1, v_2; \mathcal{E}) - \max_i f(v_1, v_2; \mathcal{E}_i). \end{aligned} \quad \text{(Equation 5)}$$

To link back to our explanation above, we can take the expected value of these quantities with respect to $p(\omega)$ (which in the simplest case is an average across all pairs of nodes). This yields the average quantities $R(\mathcal{M}) = \mathbb{E}[r(\Omega; \mathcal{M})]$ and similarly for unique and synergistic contributions. With this, we can write:

$$F(\mathcal{E}) = R(\mathcal{M}) + U_1(\mathcal{M}) + U_2(\mathcal{M}) + S(\mathcal{M}), \quad \text{(Equation 6)}$$

proving that, indeed, we have decomposed average global efficiency into four constituent quantities. Note that the global efficiency depends only on the joint network but each atom depends on the full multilayer network, since they depend on which edges are in $\mathcal{E}_1$ or $\mathcal{E}_2$.

To summarize the overall prevalence of synergistic, unique, and redundant paths in a network, we can define the dominant character of a given node pair $\omega = (v_1, v_2)$ as the highest-order non-zero atom in its PND. For example, in the two-layer case described here, we say that ω is a synergistic pair if $s(\omega; \mathcal{M}) > 0$, or a unique pair if $u_j(\omega; \mathcal{M}) > 0$ and $s(\omega; \mathcal{M}) = 0$ (for any j), or a redundant pair if $r(\omega; \mathcal{M}) > 0$ and $s(\omega; \mathcal{M}) = u_j(\omega; \mathcal{M}) = 0$ (for all j).

General framework of partial network decomposition

After presenting an elementary example, let us introduce our full formalism, PND: an approach to multilayer network analysis inspired by PID.[18]

Consider a set of nodes $\mathcal{V}$ and N sets of edges $\{\mathcal{E}_i\}_{i=1}^N$, such that the tuples $(\mathcal{V}, \mathcal{E}_i)$ form networks with the same nodes but different edges. For a given set of indices $a = \{n_1, .., n_k\} \subseteq \{1, \ldots, N\}$, we define the joint network $(\mathcal{V}, \mathcal{E}^a)$ with $\mathcal{E}^a = \cup_{i=1}^k \mathcal{E}_{n_i}$. Furthermore, let us denote collections of such networks by $\alpha = \{a_1, \ldots, a_L\}$. For example, possible collections of networks for $n = 2$ are $\{\varnothing\}, \{\{1\}\}, \{\{1\}, \{1,2\}\}$, etc.

The key quantity in PND is the "network redundancy function" $F_\cap(\mathcal{E}^{a_1}, \mathcal{E}^{a_2}, \ldots, \mathcal{E}^{a_L})$, which we will also denote with the shorthand notation $F_\cap^\alpha$.

This function should capture how much of the value of F is due to the "common contribution" of all networks $\mathcal{E}^{a_1}, \ldots, \mathcal{E}^{a_L}$. We require this function to have the following properties:

Symmetry: $F_\cap(\mathcal{E}^{a_1}, \ldots, \mathcal{E}^{a_L})$ is invariant to reordering of $\mathcal{E}^{a_1}, \ldots, \mathcal{E}^{a_L}$.
Self-intersection: $F_\cap(\mathcal{E}^a) = F(\mathcal{E}^a)$. This links PND with the original network property of interest and is analogous to the set-theoretic statement that $S \cap S = S$.
Deterministic equality: $F_\cap(\mathcal{E}^{a_1}, \ldots, \mathcal{E}^{a_L}) = F_\cap(\mathcal{E}^{a_1}, \ldots, \mathcal{E}^{a_{L-1}})$ if $\mathcal{E}^{a_{L-1}} \subseteq \mathcal{E}^{a_L}$. This is analogous to the usual set-theoretic statement that $S \cap T = S$ if $S \subseteq T$.

In principle, one could apply $F_\cap^\alpha$ to any collection of networks $\alpha \in \mathcal{P}_1(\mathcal{P}_1(\{1,\ldots,N\}))$, where $\mathcal{P}_1(S)$ is the power set of S excluding the empty set. However, the deterministic equality axiom allows us to simplify the domain of $F_\cap$: for example, for the two-layer case we know that $F_\cap(\mathcal{E}^{\{1\}},\mathcal{E}^{\{1,2\}}) = F_\cap(\mathcal{E}^{\{1\}})$, because $\mathcal{E}^{\{1\}}$ is contained in $\mathcal{E}^{\{1,2\}}$ (recall that $\mathcal{E}^{\{1,2\}} = \mathcal{E}_1 \cup \mathcal{E}_2$). In the general case, this means we can restrict the domain of $F_\cap^\alpha$ to the set of "antichains" of $\{1,\ldots,N\}$, denoted here as $\mathcal{A}$, which are naturally organized in a set-theoretic construct known as a "lattice."[18] For the two-layer case, the possible antichains are $\{\{1\},\{2\}\}$, $\{\{1\}\}$, $\{\{2\}\}$, and $\{\{1,2\}\}$.

Although the three properties are the only ones necessary to formulate our PND over the antichain lattice, there is one more property that will be of interest for the interpretation of the resulting decomposition:

Monotonicity: $F_\cap(\mathcal{E}^{a_1},\ldots,\mathcal{E}^{a_k}) \le F_\cap(\mathcal{E}^{a_1},\ldots,\mathcal{E}^{a_{k-1}})$.

Given a network redundancy function $F_\cap^\alpha$, one can also ask how a specific collection of networks α contributes to the overall utility of the joint network. To quantify this, we can define utility "atoms" that capture the contribution of α over and above the contribution of other elements lower in the lattice,

$$F_\partial^\alpha = F_\cap^\alpha - \sum_{\beta \prec \alpha} F_\partial^\beta, \qquad \text{(Equation 7)}$$

where $\prec$ (and $\succeq$) correspond to the natural partial order in the antichain lattice.[18] This is equivalent to saying that F_∂^α is the Moebius inversion of $F_\cap^\alpha$[18] and can also be written as:

$$F_\cap^\alpha = \sum_{\beta \preceq \alpha} F_\partial^\beta, \qquad \text{(Equation 8)}$$

which, together with the self-intersection property, also implies that the sum of all atoms decomposes F as expected:

$$F(\mathcal{E}) = F_\cap^{\{\{1,\ldots,N\}\}} = \sum_{\alpha \in \mathcal{A}} F_\partial^\alpha. \qquad \text{(Equation 9)}$$

These atoms are the objects of interest in our analyses and correspond to the redundant, unique, and synergistic contributions to global efficiency presented in the previous section.

In addition to the formalism above, we need one more ingredient to compute these atoms: a definition of the network redundancy function $F_\cap$. Although more definitions satisfying the properties above could certainly be possible, here we propose the following definition for its suitability and simplicity:

$$F_\cap^\alpha = \mathbb{E}\left[\min_{a \in \alpha} f(\Omega; \mathcal{E}^a)\right]. \qquad \text{(Equation 10)}$$

With Ω defined as above and f being the efficiency (inverse of the shortest path length), it is easy to see that this definition satisfies the symmetry, self-intersection, and monotonicity axioms above. Furthermore, it is possible to prove that this definition of $F_\cap^\alpha$ is *totally monotone* in $\mathcal{A}$, which guarantees that all network atoms F_∂^α are non-negative (see proofs in the supplemental experimental procedures). With this definition, it is possible to evaluate Equation 10 on all antichains and then use Equation 7 to compute all atoms, concluding the calculation.

It is worth noting that, in analogy with the previous section, we can naturally define a redundancy function for node pairs ω, i.e., $f_\cap^\alpha(\omega) = \min_{a \in \alpha} f(\omega; \mathcal{E}^a)$, such that

$F_{\cap}^{\alpha} = \mathbb{E}[f_{\cap}^{\alpha}(\omega)]$ (and similarly for $f_{\partial}^{\alpha}(\omega)$). With this, we can directly generalize our previous definition of the dominant character of a node pair ω as the set α such that $f_{\partial}^{\alpha}(\omega) > 0$ and $f_{\partial}^{\beta}(\omega) = 0 \forall \beta \succ \alpha$. It is direct to show that there is always a unique α satisfying this condition for $N = 2$, and numerical experiments suggest this is also the case for $N > 2$—although, in the absence of a proof, we leave this as a conjecture for future work.

In addition to a choice of utility function F and redundancy function $F_{\cap}$, PND also allows the choice of different distributions $p(\omega)$. For simplicity, here we consider only a uniform distribution as a way to obtain a simple average of the decomposition across the network. Nonetheless, different choices may be more or less useful in different contexts, depending greatly on the networks under study and the specific scientific goals of the analysis—for example, one could consider a $p(\omega)$ that takes into account some measure of centrality or some aspect of the real-world usage of the network (e.g., number of passengers in transit between two locations).

Finally, it is worth noticing that—in contrast with PID—PND decomposes the effect of various source networks but does not consider a target. This technical difference has interesting implications. For example, PND does not have an "identity axiom" (an apparently reasonable requirement,[41] which nonetheless has been shown to be incompatible with non-negativity[42]) and is exempt from the constraints that information decomposition must satisfy under deterministic relationships between sources and target.[43] The investigation of these and other implications of the PND formalism are left for future work.

Data

London transport networks

The network of London public rail transport was obtained from https://networks.skewed.de/net/london_transport. Further details are available from the original publication by De Domenico et al.[44] It is a multiplex network with three undirected edge types representing links within the three layers of London train stations: underground, overground, and DLR (Docklands Light Railway). Here, we combined overground and DLR into a single network, and we then compared the respective contributions of underground vs. overground + DLR (which we refer to as simply "overground").

Human structural connectomes from the Human Connectome Project

We used diffusion MRI (dMRI) data from the 100 unrelated subjects (54 females and 46 males, mean age 29.1 ± 3.7 years) of the HCP 900 subjects data release.[45] All HCP scanning protocols were approved by the local institutional review board at Washington University in St. Louis. The diffusion-weighted imaging (DWI) acquisition protocol is covered in detail elsewhere.[46] The diffusion MRI scan was conducted on a Siemens 3T Skyra scanner using a 2D spin-echo single-shot multiband EPI sequence with a multiband factor of 3 and monopolar gradient pulse. The spatial resolution was 1.25 mm isotropic; TR = 5,500 ms, TE = 89.50 ms. The b values were 1,000, 2,000, and 3,000 s/mm^2. The total numbers of diffusion sampling directions were 90, 90, and 90 for each of the shells in addition to six b0 images. We used the version of the data made available in DSI Studio-compatible format at http://brain.labsolver.org/diffusion-mri-templates/hcp-842-hcp-1021.[47]

We adopted previously reported procedures to reconstruct the human connectome from DWI data. The minimally preprocessed DWI HCP data[46] were corrected for

eddy current and susceptibility artifact. DWI data were then reconstructed using q-space diffeomorphic reconstruction (QSDR[48]), as implemented in DSI Studio (www.dsi-studio.labsolver.org). QSDR is a model-free method that calculates the orientation distribution of the density of diffusing water in a standard space to conserve the diffusible spins and preserve the continuity of fiber geometry for fiber tracking. QSDR first reconstructs diffusion-weighted images in native space and computes the quantitative anisotropy (QA) in each voxel. These QA values are used to warp the brain to a template QA volume in Montreal Neurological Institute (MNI) space using a non-linear registration algorithm implemented in the statistical parametric mapping (SPM) software. A diffusion sampling length ratio of 2.5 was used, and the output resolution was 1 mm. A modified FACT (fiber assignment by continuous tracking) algorithm[49] was then used to perform deterministic fiber tracking on the reconstructed data, with the following parameters[36]: angular cutoff of 55°, step size of 1.0 mm, minimum length of 10 mm, maximum length of 400 mm, spin density function smoothing of 0.0, and a QA threshold determined by DWI signal in the cerebrospinal fluid (CSF). Each of the streamlines generated was automatically screened for its termination location. A white-matter mask was created by applying DSI Studio's default anisotropy threshold (0.6 Otsu's threshold) to the spin distribution function's anisotropy values. The mask was used to eliminate streamlines with premature termination in the white-matter region. Deterministic fiber tracking was performed until $1,000,000$ streamlines were reconstructed for each individual.

For each individual, their structural connectome was reconstructed by drawing an edge between each pair of regions from the Schaefer-200 cortical atlas[50] if there were white-matter tracts connecting the corresponding brain regions end-to-end; edge weights were quantified as the number of streamlines connecting each pair of regions.

A consensus matrix A across subjects (consensus connectome) was then obtained using the procedure of Wang and colleagues[51] as follows: for each pair of regions i and j, if more than half of the subjects had a non-zero connection between i and j, A_{ij} was set to the average across all subjects with non-zero connections between i and j. Otherwise, A_{ij} was set to zero.

Alternative structural connectome from Lausanne dataset

A total of N = 70 healthy participants (25 females, age 28.8 ± 8.9 years) were scanned at the Lausanne University Hospital in a 3T MRI scanner (Trio, Siemens Medical, Germany) using a 32-channel head coil.[52] Informed written consent was obtained for all participants in accordance with institutional guidelines, and the protocol was approved by the Ethics Committee of Clinical Research of the Faculty of Biology and Medicine, University of Lausanne, Switzerland. The protocol included: (1) a magnetization-prepared rapid-acquisition gradient echo (MPRAGE) sequence sensitive to white/gray matter contrast (1 mm in-plane resolution, 1.2 mm slice thickness) and (2) a diffusion spectrum imaging (DSI) sequence (128 diffusion-weighted volumes and a single b0 volume, maximum b value $8,000$ s/mm^2, $2.2 \times 2.2 \times 3.0$ mm voxel size).

Structural connectomes were reconstructed for individual participants using deterministic streamline tractography and divided according to a subdivision of the Desikan-Killiany anatomical parcellation, with 234 cortical and subcortical regions (chosen as the closest match for the 200-node Schaefer parcellation). White matter and gray matter were segmented from the MPRAGE volumes using the FreeSurfer

(version 5.0.0) open-source package, whereas DSI data preprocessing was implemented with tools from the Connectome Mapper open-source software, initiating 32 streamline propagations per diffusion direction for each white-matter voxel. Structural connectivity was defined as streamline density between node pairs, i.e., the number of streamlines between two regions normalized by the mean length of the streamlines and the mean surface area of the regions, following previous work with these data.[34,53]

Human functional connectomes

We used resting-state functional MRI (rs-fMRI) data from the same 100 unrelated subjects of the HCP 900 subjects data release.[45] The following sequences were used: for structural MRI, 3D MPRAGE T1-weighted, TR = 2,400 ms, TE = 2.14 ms, TI = 1,000 ms, flip angle 8°, FOV 224 × 224, voxel size 0.7 mm isotropic, and for two sessions of 15-min rs-fMRI, gradient-echo EPI, TR = 720 ms, TE = 33.1 ms, flip angle 52°, FOV 208 × 180, voxel size 2 mm isotropic. Here, we used functional data from only the first scanning session, in the LR (left-right) direction.

Functional MRI denoising. We used the minimally preprocessed fMRI data from the HCP, which includes bias field correction, functional realignment, motion correction, and spatial normalization to MNI-152 standard space with 2 mm isotropic resampling resolution.[46] We also removed the first 10 volumes to allow magnetization to reach steady state. Additional denoising steps were performed using the SPM12-based toolbox CONN (http://www.nitrc.org/projects/conn), version 17f.[54] To reduce noise due to cardiac and motion artifacts, we applied the anatomical CompCor method of denoising the functional data. The anatomical CompCor method (also implemented within the CONN toolbox) involves regressing out of the functional data the following confounding effects: the first five principal components attributable to each individual's white-matter signal, the first five components attributable to individual CSF signal, and six subject-specific realignment parameters (three translations and three rotations) as well as their first-order temporal derivatives.[54] Linear detrending was also applied, and the subject-specific denoised BOLD signal time series were band-pass filtered to eliminate both low-frequency drift effects and high-frequency noise, thus retaining frequencies between 0.008 and 0.09 Hz.

Functional network reconstruction. FC networks were obtained for each subject by correlating the BOLD time series of each pair of regions in the Schaefer atlas. A group-average FC network was then obtained by averaging across all subjects. To ensure comparability of the structural and functional networks, the functional networks were each thresholded to have the same density as the corresponding structural network, a procedure termed "structural density matching."[36] Both structural and functional networks were binarized before analysis.

Mammalian connectomes from diffusion MRI

We used data available online (https://doi.org/10.5281/zenodo.7143143); below we provide the main information, with further detail available in the original publication.[28] For consistency of reporting, where possible, we use the same wording as recent publications using this dataset.[4,28]

Brain samples. The MAMI database includes a total of 220 *ex vivo* diffusion and T2- and T1-weighted brain scans of 124 different animal species. No animals were deliberately euthanized for this study. All brains were collected based on incidental death of animals in zoos in Israel, or natural death collected abroad, and with the

permission of the national park authority (approval no. 2012/38645) or its equivalent in the relevant country. All scans were performed on excised and fixated tissue. Animals' brains were extracted within 24 h of death and placed in formaldehyde (10%) for a fixation period of a few days to a few weeks (depending on the brain size). Approximately 24 h before the MRI scanning session, the brains were placed in phosphate-buffered saline for rehydration. Given the limited size of the bore, small brains were scanned using a 7T 30/70 BioSpec Avance Bruker system, whereas larger brains were scanned using a 3T Siemens Prisma system. To minimize image artifacts caused by magnet susceptibility effects, the brains were immersed in fluorinated oil (Fluorinert, 3M) inside a plastic bag during the MRI scanning session.

MRI acquisition. A unified MRI protocol was implemented for all species. The protocol included high-resolution anatomical scans (T2- or T1-weighted MRI), which were used as an anatomical reference, and diffusion MR scans. Diffusion MRI data were acquired using high-angular-resolution diffusion imaging (HARDI), which consists of a series of diffusion-weighted, spin-echo, echo-planar-imaging images covering the whole brain, scanned in either 60 (in the 7T scanner) or 64 gradient directions (in the 3T scanner) with an additional three non-diffusion-weighted images (b0). The b value was 1,000 s/mm^2 in all scans. In the 7T scans, TR was longer than $12,000$ ms (depending on the number of slices), with TE of 20 ms. In the 3T scans, TR was $3,500$ ms, with a TE of 47 ms.

To linearly scale according to brain size the 2D image pixel resolution (per slice), the size of the matrix remained constant across all species (128×96). Due to differences in brain shape, the number of slices varied between 46 and 68. Likewise, the number of scan repetitions and the acquisition time were different for each species, depending on brain size and desired signal-to-noise ratio (SNR) levels. To keep SNR levels above 20, an acquisition time of 48 h was used for small brains ($\sim$0.15 mL) and 25 min for large brains ($>1,000$ mL). SNR was defined as the ratio of mean signal strength to the standard deviation of the noise (an area in the non-brain part of the image). Full details are provided in the original publication.[28]

Connectome reconstruction. The ExploreDTI software was used for diffusion analysis and tractography.[55] The following steps were used to reconstruct fiber tracts. To reduce noise and smooth the data, anisotropic smoothing with a 3-pixel Gaussian kernel was applied. Motion, susceptibility, and eddy current distortions were corrected in the native space of the HARDI acquisition. A spherical harmonic deconvolution approach was used to generate fiber-orientation density functions per pixel, yielding multiple ($n \geq 1$) fiber orientations per voxel. Spherical harmonics of up to fourth order were used.[56] Whole-brain tractography was performed using a constrained spherical deconvolution (CSD) seed point threshold similar for all samples (0.2) and a step length half the pixel size. The end result of the tractography analysis was a list of streamlines starting and ending between pairs of voxels. Recent studies have shown that fiber tracking tends to present a bias where the vast majority of endpoints reside in the white matter.[56] To avoid this, the CSD tracking implemented here ensures that approximately 90% of the endpoints reside in the cortical and subcortical gray matter.

Network reconstruction. Before the reconstruction of the structural networks, certain fiber tracts were removed from the final list of tracts. These include external projection fibers that pass through the cerebral peduncle, as well as cerebellar connections. Inner-hemispheric projections, such as the thalamic radiation, were

included in the analysis. Brains were parcellated into 200 nodes using a k-means clustering algorithm. All the fiber endpoint positions were used as input, and cluster assignment was done based on the similarity in connectivity profile between pairs of endpoints. Therefore, vertices with similar connectivity profiles have a higher chance of grouping together. The clustering was performed twice, once for each hemisphere. Nodes were defined as the center of mass of the resulting 200 clusters. Connectivity matrices were generated by counting the number of streamlines between any two nodes.[28] The resulting connectivity matrices were hence sparse and weighted adjacency matrices. Matrices were binarized by setting connectivity values to 1 if the connection existed and 0 otherwise.

Even though the sizes of the regions differ across species, we opted for a uniform parcellation scheme (i.e., 200 nodes) for several reasons, in keeping with previous work.[4] First, to our knowledge, there is no MRI parcellation for the brains of the majority of the species studied here. Second, how brain regions correspond to one another across species (i.e., homologs) is still not completely understood for many regions and for many species. Third, comparing networks of different sizes introduces numerous analytical biases because most network measures trivially depend on size, making the comparison challenging. We therefore opted to implement a uniform parcellation scheme across species, allowing us to translate connectomes into a common reference feature space in which they can be compared. Note that this approach does not take into account species-specific regional delineations, nor does it capture homologies between nodes across species, which are still not completely understood.

Identification of long- and short-range connections

For both human structural and functional networks, as well as mammalian structural networks, connection length was defined in terms of Euclidean distance between the centroids of the regions of interest constituting the endpoints of each edge.

A short-range (or respectively long-range) connectivity network was obtained as the shortest (longest) 50% of edges. Thus, for each starting network, we obtained a network of its 50% shortest edges and a network of its 50% longest edges. This approach ensures that both networks have equal density and are therefore on equal terms in terms of their expected contribution to the composite network.

Network models

Binary, undirected Erdős-Rényi networks of different densities were created by randomly selecting a fraction f of all possible bidirectional edges in the network and setting them to unity, with f ranging between 0.01 and 1.0 in increments of 0.01.

For the rewiring experiment, we initially generated a binary, undirected network of 200 nodes with lattice topology and 5% density. A copy of this network was then generated, and its edges were progressively randomized in 1% increments, using a standard rewiring procedure to preserve the degree of each node.

Small-world character was measured via the small-world propensity (SWP) index,[24] which quantifies the deviation of the network's empirically observed clustering coefficient, C_{obs}, and characteristic path length, L_{obs}, from equivalent lattice (C_{latt}, L_{latt}) and random (C_{rand}, L_{rand}) networks of equal numbers of nodes and edges:

$$SWP = 1 - \sqrt{\left(\Delta_C^2 + \Delta_L^2\right)/2} \quad \text{(Equation 11)}$$

with

$$\Delta_C = (C_{latt} - C_{obs}) / (C_{latt} - C_{rand}) \quad \text{(Equation 12)}$$

and

$$\Delta_L = (L_{obs} - L_{rand}) / (L_{latt} - L_{rand}), \quad \text{(Equation 13)}$$

such that SWP is bound between 0 and 1.

To disambiguate the role of connectome topology in shaping the contribution to shortest paths, we relied on network null models.[57] Specifically, we adopted two types of null models: the well-known Maslov-Sneppen degree-preserving rewired network, whereby edges are swapped so as to randomize the topology while preserving the exact binary degree of each node (degree sequence), which therefore also implies preserving the network's density,[58] and a more stringent geometry-preserving null model that, in addition to the degree sequence, also approximately preserves the edge-length distribution of the original network (based on Euclidean distance between regions).[27]

Statistical analysis

For the London transport networks, statistical significance of the empirical results was assessed against a population of 1,000 network null models, constructed as described above. For both human and animal brain networks, the null distribution was obtained by rewiring each individual network, and the empirical and null distributions were compared using permutation-based paired t tests (with 10,000 permutations). Non-parametric tests were chosen to ensure robustness to outliers. Effect size was computed as Hedge's *g*. Tables with full descriptive statistics for the results reported in the main text are provided as supplemental tables.

SUPPLEMENTAL INFORMATION

Supplemental information can be found online at https://doi.org/10.1016/j.xcrp.2024.101892.

ACKNOWLEDGMENTS

This research was supported by the Visitor Program of the Max Planck Institute for Mathematics in the Sciences, Leipzig (Germany) (to A.I.L.), and a Gates Cambridge Scholarship (OPP 1144) (to A.I.L.). F.E.R. was supported by the Fellowship Programme of the Institute of Cultural and Creative Industries of the University of Kent and the DIEP visitors program at the University of Amsterdam. J.J. acknowledges support from grant 1514 of the German Israeli Foundation. The authors are grateful to Yaniv Assaf and the Strauss Center for Neuroimaging of Tel Aviv University for making the MAMI dataset available; to Joe Lizier, Pradeep Banerjee, and members of the Network Neuroscience Lab at McGill University for helpful discussion; and to anonymous peer reviewers for helpful suggestions.

AUTHOR CONTRIBUTIONS

A.I.L. conceived the research. A.I.L., E.O., F.E.R., P.A.M.M., C.F., and J.J. designed the analysis methodology. F.E.R. and P.A.M.M. developed the mathematical formalism. A.I.L. analyzed the data and generated figures. L.E.S. contributed to data processing and visualization. A.I.L., F.E.R., and P.A.M.M. wrote the first draft of the manuscript with input and feedback from all coauthors.

DECLARATION OF INTERESTS

The authors declare no competing interests.

Received: November 14, 2023
Revised: January 18, 2024
Accepted: March 1, 2024
Published: March 28, 2024

REFERENCES

1. Gao, X., Xiao, B., Tao, D., and Li, X. (2010). A survey of graph edit distance. Pattern Anal. Appl. *13*, 113–129.
2. Borgwardt, K.M., and Kriegel, H.-P. (2005). Shortest-path kernels on graphs. In Fifth IEEE International Conference on Data Mining (ICDM'05) (IEEE), p. 8.
3. de Lange, S.C., de Reus, M.A., and van den Heuvel, M.P. (2014). The Laplacian spectrum of neural networks. Front. Comput. Neurosci. *7*, 189.
4. Suarez, L.E., Yovel, Y., van den Heuvel, M.P., Sporns, O., Assaf, Y., Lajoie, G., and Misic, B. (2022). A connectomics-based taxonomy of mammals. Elife *11*, e78635.
5. De Domenico, M., Nicosia, V., Arenas, A., and Latora, V. (2015). Structural reducibility of multilayer networks. Nat. Commun. *6*, 6864.
6. De Domenico, M., and Biamonte, J. (2016). Spectral entropies as information-theoretic tools for complex network comparison. Phys. Rev. X *6*, 041062.
7. Bagrow, J.P., and Bollt, E.M. (2019). An information-theoretic, all-scales approach to comparing networks. Appl. Netw. Sci. *4*. 45–15.
8. Kawahara, J., Brown, C.J., Miller, S.P., Booth, B.G., Chau, V., Grunau, R.E., Zwicker, J.G., and Hamarneh, G. (2017). BrainNetCNN: Convolutional neural networks for brain networks; towards predicting neurodevelopment. Neuroimage *146*, 1038–1049.
9. Mheich, A., Hassan, M., Khalil, M., Gripon, V., Dufor, O., and Wendling, F. (2018). Siminet: a novel method for quantifying brain network similarity. IEEE Trans. Pattern Anal. Mach. Intell. *40*, 2238–2249.
10. Shimada, Y., Hirata, Y., Ikeguchi, T., and Aihara, K. (2016). Graph distance for complex networks. Sci. Rep. *6*, 34944–34946.
11. Schieber, T.A., Carpi, L., Díaz-Guilera, A., Pardalos, P.M., Masoller, C., and Ravetti, M.G. (2017). Quantification of network structural dissimilarities. Nat. Commun. *8*, 13928.
12. Cao, B., Li, Y., and Yin, J. (2013). Measuring similarity between graphs based on the levenshtein distance. Appl. Math. Inf. Sci. *7*, 169–175.
13. Koutra, D., Vogelstein, J.T., and Faloutsos, C. (2013). Deltacon: A principled massive-graph similarity function. In Proceedings of the 2013 SIAM International Conference on Data Mining (SIAM), pp. 162–170.
14. Lacasa, L., Stramaglia, S., and Marinazzo, D. (2021). Beyond pairwise network similarity: exploring mediation and suppression between networks. Commun. Phys. *4*, 136.
15. Wills, P., and Meyer, F.G. (2020). Metrics for graph comparison: a practitioner's guide. PLoS One *15*, e0228728.
16. Tantardini, M., Ieva, F., Tajoli, L., and Piccardi, C. (2019). Comparing methods for comparing networks. Sci. Rep. *9*, 17557–17619.
17. Ahmad, M., Wendling, F., and Hassan, M. (2020). Brain network similarity: methods and applications. Network Neuroscience *4*, 507–527.
18. P.L. Williams and R.D. Beer. Nonnegative decomposition of multivariate information. Preprint at: arXiv. 10.48550/arXiv.1004.2515
19. P.A.M. Mediano, F.E. Rosas, A.I. Luppi, R.L. Carhart-Harris, D. Bor, A.K. Seth, and A.B. Barrett. Towards an Extended Taxonomy of Information Dynamics via Integrated Information Decomposition. Preprint at: arXiv. 10.48550/arXiv.2109.13186
20. Luppi, A.I., Mediano, P.A.M., Rosas, F.E., Holland, N., Fryer, T.D., O'Brien, J.T., Rowe, J.B., Menon, D.K., Bor, D., and Stamatakis, E.A. (2022). A synergistic core for human brain evolution and cognition. Nat. Neurosci. *25*, 771–782.
21. Luppi, A.I., Rosas, F.E., Mediano, P.A.M., Menon, D.K., and Stamatakis, E.A. (2024). Information decomposition and the informational architecture of the brain. Trends Cognit. Sci. https://doi.org/10.1016/j.tics.2023.11.005.
22. Di Lanzo, C., Marzetti, L., Zappasodi, F., De Vico Fallani, F., and Pizzella, V. (2012). Redundancy as a graph-based index of frequency specific meg functional connectivity. Comput. Math. Methods Med. *2012*, 207305.
23. Watts, D.J., and Strogatz, S.H. (1998). Collective dynamics of 'small-world' networks. Nature *393*, 440–442.
24. Muldoon, S.F., Bridgeford, E.W., and Bassett, D.S. (2016). Small-world propensity and weighted brain networks. Sci. Rep. *6*, 22057.
25. Suárez, L.E., Markello, R.D., Betzel, R.F., and Misic, B. (2020). Linking structure and function in macroscale brain networks. Trends Cogn. Sci. *24*, 302–315. https://doi.org/10.1016/j.tics.2020.01.008.
26. Betzel, R.F., Griffa, A., Hagmann, P., and Mišić, B. (2019). Distance-dependent consensus thresholds for generating group-representative structural brain networks. Netw. Neurosci. *3*, 475–496. https://doi.org/10.1162/netn_a_00075.
27. Betzel, R.F., and Danielle, S.B. (2018). Specificity and Robustness of Long-Distance Connections in Weighted, Interareal Connectomes. Proc. Natl. Acad. Sci. USA *115*, E4880–E4889. https://doi.org/10.1073/pnas.1720186115.
28. Assaf, Y., Bouznach, A., Zomet, O., Marom, A., and Yovel, Y. (2020). Conservation of brain connectivity and wiring across the mammalian class. Nat. Neurosci. *23*, 805–808.
29. Seguin, C., Van Den Heuvel, M.P., and Zalesky, A. (2018). Navigation of brain networks. Proc. Natl. Acad. Sci. USA *115*, 6297–6302.
30. Seguin, C., Razi, A., and Zalesky, A. (2019). Inferring neural signalling directionality from undirected structural connectomes. Nat. Commun. *10*, 4289.
31. Avena-Koenigsberger, A., Misic, B., and Sporns, O. (2017). Communication dynamics in complex brain networks. Nat. Rev. Neurosci. *19*, 17–33. https://doi.org/10.1038/nrn.2017.149.
32. Seguin, C., Sporns, O., and Zalesky, A. (2023). Brain network communication: concepts, models and applications. Nat. Rev. Neurosci. *24*, 557–574.
33. Danielle, S. (2017). Bassett and Olaf Sporns. Network neuroscience. Nat. Neurosci. *20*, 353–364. https://doi.org/10.1038/nn.4502.
34. Vázquez-Rodríguez, B., Suárez, L.E., Markello, R.D., Shafiei, G., Paquola, C., Hagmann, P., Van Den Heuvel, M.P., Bernhardt, B.C., Spreng, R.N., and Misic, B. (2019). Gradients of structure–function tethering across neocortex. Proc. Natl. Acad. Sci. USA *116*, 21219–21227.
35. Guy, M. (2006). Just how dense are dense graphs in the real world? a methodological note. In Proceedings of the 2006 AVI Workshop on beyond Time and Errors: Novel Evaluation Methods for Information Visualization, pp. 1–7.
36. Luppi, A.I., and Stamatakis, E.A. (2021). Combining network topology and information theory to construct representative brain networks. Netw. Neurosci. *5*, 96–124.
37. Stanford, W.C., Mucha, P.J., and Dayan, E. (2022). A robust core architecture of functional brain networks supports topological resilience and cognitive performance in middle-and old-aged adults. Proc. Natl. Acad. Sci. USA *119*, e2203682119.
38. Harush, U., and Barzel, B. (2017). Dynamic patterns of information flow in complex networks. Nat. Commun. *8*, 2181.

39. Kirst, C., Timme, M., and Battaglia, D. (2016). Dynamic information routing in complex networks. Nat. Commun. *7*, 11061.

40. Dietrich, B. (1968). Über ein paradoxon aus der verkehrsplanung. Unternehmensforschung *12*, 258–268.

41. Harder, M., Salge, C., and Polani, D. (2013). Bivariate measure of redundant information. Phys. Rev. *87*, 012130.

42. Rauh, J., Bertschinger, N., Olbrich, E., and Jost, J. (2014). Reconsidering unique information: Towards a multivariate information decomposition. In 2014 IEEE International Symposium on Information Theory (IEEE), pp. 2232–2236.

43. Chicharro, D., Pica, G., and Panzeri, S. (2018). The identity of information: how deterministic dependencies constrain information synergy and redundancy. Entropy *20*, 169.

44. De Domenico, M., Solé-Ribalta, A., Gómez, S., and Arenas, A. (2014). Navigability of interconnected networks under random failures. Proc. Natl. Acad. Sci. USA *111*, 8351–8356.

45. Van Essen, D.C., Smith, S.M., Barch, D.M., Behrens, T.E.J., Yacoub, E., and Ugurbil, K.; WU-Minn HCP Consortium (2013). The wu-minn human connectome project: An overview. Neuroimage *80*, 62–79. https://doi.org/10.1016/j.neuroimage.2013.05.041.

46. Glasser, M.F., Sotiropoulos, S.N., Wilson, J.A., Coalson, T.S., Fischl, B., Andersson, J.L., Xu, J., Jbabdi, S., Webster, M., Polimeni, J.R., et al. (2013). The minimal preprocessing pipelines for the human connectome project. Neuroimage *80*, 105–124. https://doi.org/10.1016/j.neuroimage.2013.04.127.

47. Yeh, F.-C., Panesar, S., Fernandes, D., Meola, A., Yoshino, M., Fernandez-Miranda, J.C., Vettel, J.M., and Verstynen, T. (2018). Population-averaged atlas of the macroscale human structural connectome and its network topology. Neuroimage *178*, 57–68. https://doi.org/10.1016/j.neuroimage.2018.05.027.

48. Yeh, F.-C., Wedeen, V.J., and Tseng, W.Y.I. (2011). Estimation of fiber orientation and spin density distribution by diffusion deconvolution. Neuroimage *55*, 1054–1062. https://doi.org/10.1016/J.NEUROIMAGE.2010.11.087.

49. Yeh, F.-C., Verstynen, T.D., Wang, Y., Fernández-Miranda, J.C., and Tseng, W.Y.I. (2013). Deterministic diffusion fiber tracking improved by quantitative anisotropy. PLoS One *8*, 80713. https://doi.org/10.1371/journal.pone.0080713.

50. Schaefer, A., Kong, R., Gordon, E.M., Laumann, T.O., Zuo, X.N., Holmes, A.J., Eickhoff, S.B., and Yeo, B.T.T. (2018). Local-global parcellation of the human cerebral cortex from intrinsic functional connectivity mri. Cereb. Cortex *28*, 3095–3114.

51. Wang, P., Kong, R., Kong, X., Liégeois, R., Orban, C., Deco, G., Van Den Heuvel, M.P., and Thomas Yeo, B.T. (2019). Inversion of a large-scale circuit model reveals a cortical hierarchy in the dynamic resting human brain. Sci. Adv. *5*, eaat7854–12. https://doi.org/10.1126/sciadv.aat7854.

52. Griffa, A., Alemán-Gómez, Y., and Hagmann, P. (2019). Structural and Functional Connectome from 70 Young Healthy Adults [data Set] (Zenodo).

53. Hagmann, P., Cammoun, L., Gigandet, X., Meuli, R., Honey, C.J., Wedeen, V.J., and Sporns, O. (2008). Mapping the structural core of human cerebral cortex. PLoS Biol. *6*, 159. https://doi.org/10.1371/journal.pbio.0060159.

54. Whitfield-Gabrieli, S., and Nieto-Castanon, A. (2012). CONN: A functional connectivity toolbox for correlated and anticorrelated brain networks. Brain Connect. *2*, 125–141.

55. Leemans, A., Ben, J., Jan, S., and Derek, K.J. (2009). ExploreDTI: A graphical toolbox for processing, analyzing, and visualizing diffusion mr data. In Proceedings of the International Society for Magnetic Resonance in Medicine, *17*, p. 3537.

56. Tournier, J.D., Calamante, F., Gadian, D.G., and Connelly, A. (2004). Direct estimation of the fiber orientation density function from diffusion-weighted mri data using spherical deconvolution. Neuroimage *23*, 1176–1185.

57. Váša, F., and Mišić, B. (2022). Null models in network neuroscience. Nat. Rev. Neurosci. *23*, 493–504.

58. Maslov, S., and Sneppen, K. (2002). Specificity and stability in topology of protein networks. Science *296*, 910–913. https://doi.org/10.1126/science.1065103.

Cell Reports Physical Science, Volume *5*

Supplemental information

Quantifying synergy and redundancy between networks

Andrea I. Luppi, Eckehard Olbrich, Conor Finn, Laura E. Suárez, Fernando E. Rosas, Pedro A.M. Mediano, and Jürgen Jost

Supplemental Items

Supplemental Experimental Procedures

Alternative possible formulation of network redundancy for shortest paths

Throughout the paper, we have seen that the PND framework applied to global efficiency can quantify redundant, unique or synergistic contributions from path lengths alone. As natural as this seems, there are certain cases where this choice might clash with our intuitions for what each of these contributions should be. As an example, consider the shortest path between nodes 1 and 5 in the following two scenarios:

1. In network A_1 the shortest path is $1-2-3-5$. In network B_1 the shortest path is $1-2-3-4-5$. In $A_1 \cup B_1$ the shortest path is $1-2-3-5$.

2. In network A_2 the shortest path is $1-2-6-5$. In network B_2 the shortest path is $1-2-3-4-5$. In $A_2 \cup B_2$ the shortest path is $1-2-6-5$.

In both cases there is a unique contribution from A_1 with a path of length 4, so our current formulation considers these two cases as equivalent. Intuitively, however, some might argue that there is a sense in which A_2 is "more unique" (with respect to B_2) than A_1, because in A_2 the existence of edges $2-6$ and $6-5$ is unique and is what enables the shortest path, while in A_1 only the existence of $3-5$ is unique.

As another example where intuitions may differ, consider the shortest path between nodes 1 and 4 in the following two scenarios:

1. Network A_1 has edges $2-3-4$, but not $1-2$, and network B_1 has edges $1-2-3$, but not $3-4$. Suppose that in both networks there are other paths from 1 to 4 of length 5. Network $A_1 \cup B_1$ has a path $1-2-3-4$ which is of length 4.

2. Network A_2 has edges $3-4$, but not $1-2-3$, and network B_2 has edges $1-2-3$, but not $3-4$. Again, suppose that in both networks there are other paths from 1 to 4 of length 5. Network $A_2 \cup B_2$ has a path $1-2-3-4$ which is of length 4.

In this case, some might argue that A_2 and B_2 are "more synergistic" than A_1 and B_1 are, because in the second scenario, $2-3$ is common to both networks. But this is not reflected in our approach based on the efficiency gain provided by $A \cup B$ because in both cases you have $l_{A\cup B} = 4$ and $l_A = l_B = 5$.

Note however that these examples do not disagree that there is unique path from node 1 to node 5 in network A (for the first example), nor that there is synergy between networks A and B in the second example. The disagreement is not about the presence of uniqueness, or synergy, but only about how to quantify their extent. In other words, in terms of the three-question summary of our approach ("(1) Is there synergy? (2) If not, is there uniqueness? (3) If there is synergy or uniqueness, how much of it is there?") these examples agree on the answer to questions (1) and (2), and the divergence of intuitions only applies for question (3).

A natural alternative to the operationalisation of path redundancy that may address this concern would be one whereby shortest paths in networks A and B are redundant if and only if they involve traversing exactly the same edges (note that the two criteria coincide, for every shortest path, when network B is equal to network A). This "redundancy as identity" means that the same path would literally be available on both networks. However, we can easily see that this alternative definition of path redundancy suffers from a number of drawbacks. Mathematically, this option would be ill-defined in cases where multiple shortest paths of equal length exist, but do not coincide, with no natural way of "breaking ties". From a practical standpoint, identifying the length of the shortest paths between two nodes in networks A and B, and checking whether they are the same, is much simpler than (and a subset of) enumerating all possible shortest paths and determining whether any of them match. Finally, at a more conceptual level, in many real-world settings (e.g., travellers looking for synergies between two transport networks in going between two specific locations) the ease of reaching a destination node (in terms of time, money, or number of steps that can be saved) seems to be of far greater importance than the specific identity of the intermediate steps, or their contribution to these savings, for most purposes.

For these reasons, while we agree that there can certainly be cases where the identity and contribution of the specific sub-paths are of interest, we also believe that PND as presented is the more appealing and widely applicable alternative for the question of path efficiency (which we emphasise is only one possible application of PND) Nevertheless,we do not discard future variations of PND that take these considerations into account.

Supplemental proofs

This section contains supporting proofs for the properties of the efficiency-based Partial Network Decomposition (PND), presented in the Materials and Methods section of the main text. Our main goal is to prove that the proposed network redundancy function in Eq. (**??**) provides a non-negative PND of the network's average efficiency, such that $F^{\alpha}_{\partial} \geq 0, \forall \alpha$. We will do so by closely following the proofs for PID in Appendix D of Williams and Beer[1], bearing in mind the differences between our network redundancy function $F^{\alpha}_{\cap}$ and their information redundancy function $I_{\min}(S;\alpha)$. Note that some proofs in Ref.[1] do not depend on the properties of $I_{\min}(S;\alpha)$, and rely only on the structure of the antichain lattice —

which is shared between PID and PND. For further detail, please see the insightful discussion surrounding the non-negativity of certain PID measures in Appendix C of Ref.[2].

We begin by formally defining the efficiency f of a pair of distinct nodes $\omega = (v_1, v_2) \in \mathcal{V} \times \mathcal{V}$ s.t. $v_1 \neq v_2$ as the minimum length of all paths between them in the set of edges $\mathcal{E}$:

$$f(\omega; \mathcal{E}) = \min_{\mathtt{p} \in \mathtt{P}(\omega;\mathcal{E})} |\mathtt{p}|^{-1} \,, \tag{S1}$$

where $\mathtt{P}(\omega; \mathcal{E})$ is the set of all paths between v_1 and v_2 in $\mathcal{E}$. For convenience, we repeat the definition of the redundancy function presented in the main text,

$$f_\cap^\alpha(\omega) = \min_{a \in \alpha} f(\omega; \mathcal{E}^a) \,. \tag{S2}$$

In the following, we may omit the dependence on ω for simplicity of notation. The following theorems (up to Theorem 6) hold for all ω.

Theorem 1. *$f(\omega; \mathcal{E})$ is non-negative.*

Proof. Follows from the fact that path lengths are non-zero positive integers. Following standard convention, the shortest path length between disconnected nodes is taken to be positive infinity. □

Lemma 1. *$f(\omega; \mathcal{E}^a)$ increases monotonically under subset inclusion.*

Proof. Consider $a, b \subseteq \{1, ..., N\}$, with $a \subset b$. Recall that, by definition, $\mathcal{E}^a = \bigcup_{i=1}^k \mathcal{E}_{n_i}$ for any $a = \{n_1, .., n_k\} \subseteq \{1, \ldots, N\}$. If $a \subset b$, then $\mathcal{E}^a \subseteq \mathcal{E}^b$. Since the set of paths $\mathtt{P}(\omega; \mathcal{E})$ grows with the inclusion of more edges in $\mathcal{E}$, the minimum of any function on $\mathtt{P}$ must decrease with said inclusion, and therefore its inverse must increase. Thus, if $a \subset b$ then $f(\omega; \mathcal{E}^a) \leq f(\omega; \mathcal{E}^b)$. □

Theorem 2. *$f_\cap^\alpha$ increases monotonically in the redundancy lattice.*

Proof. This proof depends only on the structure of the redundancy lattice and Lemma 1, both of which are shared between Ref.[1] and this work. Proof is exactly as in the original work, replacing $I_{\min}(S; \alpha)$ by $f_\cap^\alpha$ and $I(S = s; a)$ by $f(\omega; \mathcal{E}^a)$. □

Theorem 3. *f_∂^α can be written in closed form as*

$$f_\partial^\alpha = f_\cap^\alpha - \sum_{k=1}^{|\alpha^-|} (-1)^{k-1} \sum_{\substack{\mathcal{B} \subseteq \alpha^- \\ |\mathcal{B}|=k}} f_\cap^{\bigwedge \mathcal{B}} \,. \tag{S3}$$

Proof. This proof depends only on the structure of the redundancy lattice and Eq. (**??**) defining f_∂^α as the Moebius inversion of $f_\cap^\alpha$, both of which are shared between Ref.[1] and this work. Proof is exactly as in the original work, replacing $I_{\min}(S; \alpha)$ by $f_\cap^\alpha$ and $\Pi_{\mathbf{R}}(S; \alpha)$ by f_∂^α. □

Theorem 4. *f_∂^α can be written in closed form as*

$$f_\partial^\alpha = f_\cap^\alpha - \max_{\beta \in \alpha^-} \min_{b \in \beta} f(\omega; \mathcal{E}^b) \,. \tag{S4}$$

Proof. First we note that, for the redundancy lattice, the following statement holds:

$$\alpha \wedge \beta = \underline{\alpha \cup \beta} \,, \tag{S5}$$

where $\underline{X}$ is the set of minimal elements of a poset X (see Refs.[1,3] for a proof). Combining Eqs. (S2) and (S3) yield

$$f_\partial^\alpha = f_\cap^\alpha - \sum_{k=1}^{|\alpha^-|} (-1)^{k-1} \sum_{\substack{\mathcal{B} \subseteq \alpha^- \\ |\mathcal{B}|=k}} \min_{b \in \bigwedge \mathcal{B}} f(\omega; \mathcal{E}^b) \,, \tag{S6}$$

and by Lemma 1 and Eq.(S5),

$$= f_\cap^\alpha - \sum_{k=1}^{|\alpha^-|} (-1)^{k-1} \sum_{\substack{\mathcal{B} \subseteq \alpha^- \\ |\mathcal{B}|=k}} \min_{\beta \in \mathcal{B}} \min_{b \in \beta} f(\omega; \mathcal{E}^b) \,. \tag{S7}$$

Then, applying the maximum-minimums identity[1] we have

$$= f_\cap^\alpha - \max_{\beta \in \alpha^-} \min_{b \in \beta} f(\omega; \mathcal{E}^b) \,, \tag{S8}$$

Or, equivalently, by applying Eq. (S2) again,

$$= f_\cap^\alpha - \max_{\beta \in \alpha^-} f_\cap^\beta \,. \tag{S9}$$

□

Theorem 5. *f_∂^α is non-negative.*

Proof. If α is the infimum of $\mathcal{A}$, $f_\partial^\alpha = f_\cap^\alpha$, and $f_\partial^\alpha \geq 0$ follows from Theorem 5. If α is not the infimum of $\mathcal{A}$, $f_\partial^\alpha \geq 0$ follows directly from Theorems 2 and 4. □

Theorem 6. *F_∂^α is non-negative.*

Proof. Follows directly from Theorem 5 and the fact that $F_\partial^\alpha = \mathbb{E}\left[f_\partial^\alpha\right]$ is a weighted sum of non-negative quantities with non-negative weights. □

As noted by Chicharro and Panzeri[2], the crucial aspect of this proof (in addition to f being non-negative and monotonically increasing) is that $f_\cap^\alpha$ is defined as a minimum of a set of values, with each value associated with a set of sources. Although the overall PND framework is applicable to other utility functions f and other redundancy functions $f_\cap^\alpha$, these may not lead to a non-negative decomposition.

Supplemental figures and tables

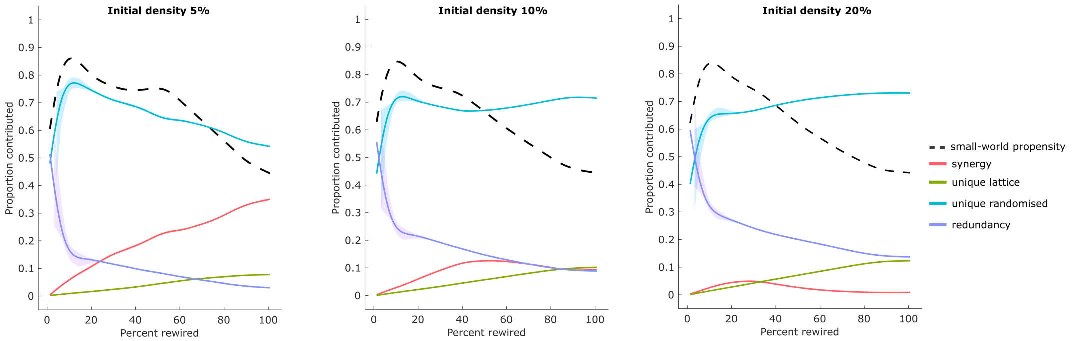

Figure S1. **Consistent effects of progressive rewiring, for networks of different density.** Synergistic, unique, and redundant contributions to global efficiency, as well as the network small-world propensity, are shown as a function of the percentage of rewired edges, for 5%, 10%, and 20% density.

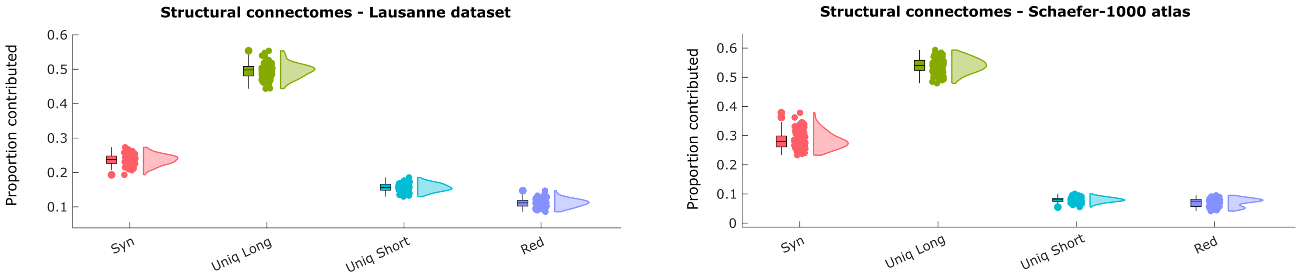

Figure S2. **Replication with alternative reconstruction of the human structural connectome**. Left: connectomes reconstructed from an independent dataset of Diffusion Spectrum Imaging data (N=70 subjects), using the Lausanne anatomical parcellation with 234 cortical and subcortical regions. Right: connectome reconstructed from an alternative sub-parcellation of the Schaefer functional atlas with 1000 cortical nodes (N=100 subjects). Y-axis: proportion of shortest paths accounted for by each PID term. Box-plots indicate the median and inter-quartile range of the distribution. Each data-point is one subject.

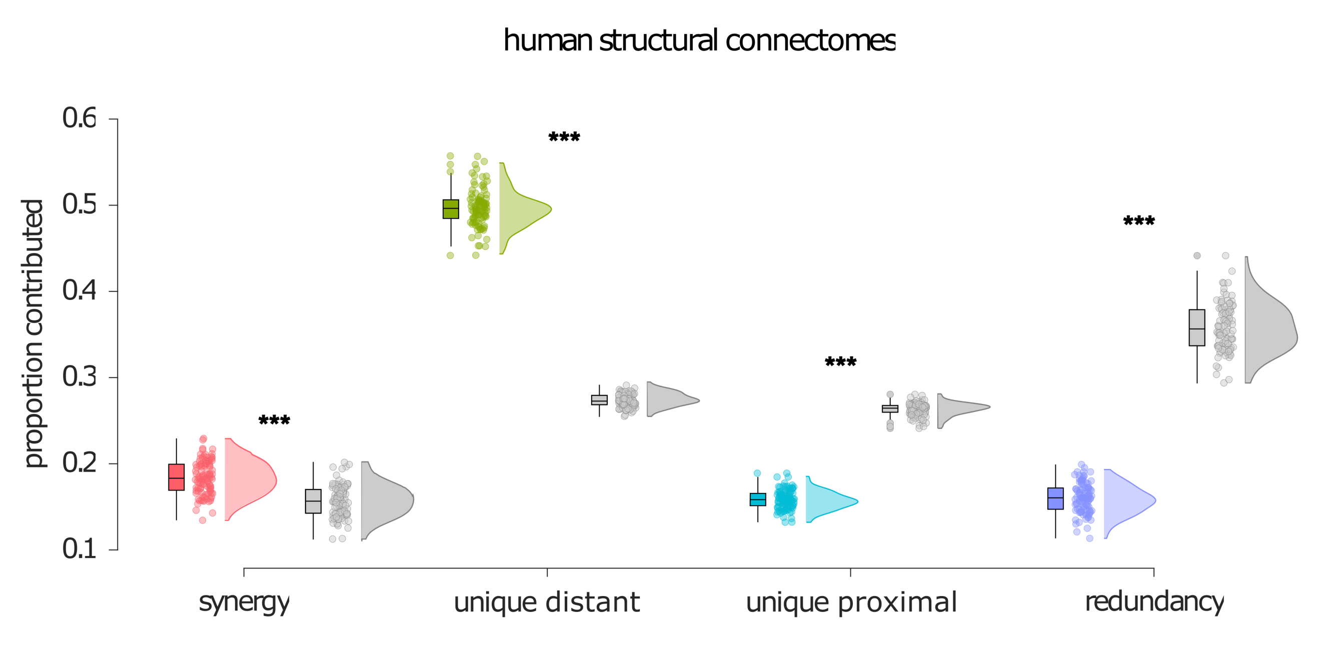

Figure S3. **Comparison against degree-preserving randomised networks**. Grey distributions indicate the corresponding values for degree-preserving randomised networks. ***: $p < 0.001$ against null distribution of values obtained from rewired null networks. Y-axis: proportion of shortest paths accounted for by each PND term. Box-plots indicate the median and inter-quartile range of the distribution. Each data-point is one subject (N=100).

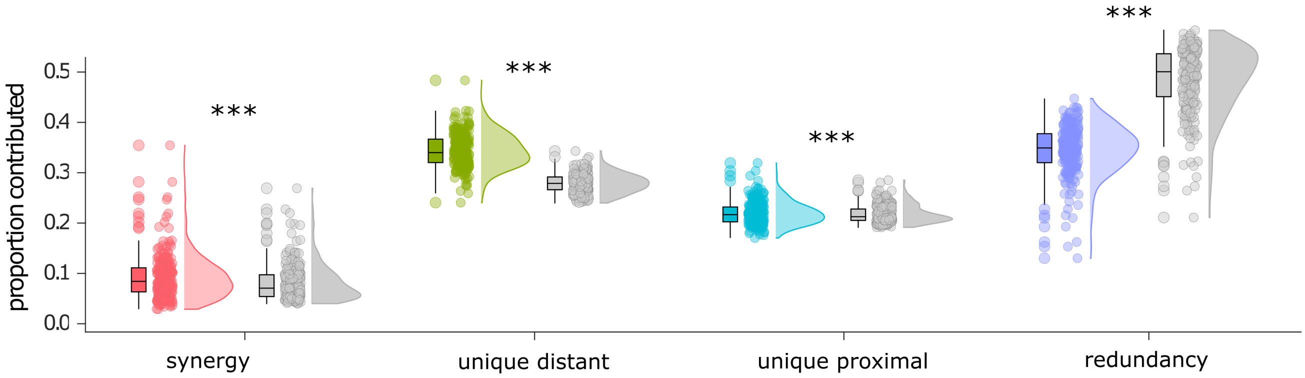

Figure S4. **Prevalence of synergistic, unique, and redundant efficiency contributions, comparing mammalian structural connectomes against degree-preserving randomised nulls.** Grey distributions indicate the corresponding values for degree- preserving randomised networks. ***: $p < 0.001$ against null distribution of values obtained from rewired null networks. Y-axis: proportion of shortest paths accounted for by each PND term. Box-plots indicate the median and inter-quartile range of the distribution. Each data-point is one animal (N=220, colored dots), or its corresponding null network (grey dots).

Contrast	Empirical Mean	Empirical SD	Null Mean	Null SD	tStat	df	pVal	Hedges g	95% CI Lower	95%CI Upper
**Synergy**	1.84e-01	1.94e-02	1.77e-01	2.08e-02	5.04	99	p <0.001	0.34	0.21	0.50
**Unique distant**	4.97e-01	2.15e-02	5.06e-01	1.16e-02	-5.53	99	p <0.001	-0.50	-0.72	-0.31
**Unique proximal**	1.58e-01	1.10e-02	1.59e-01	6.14e-03	-1.04	99	0.303	-0.10	-0.31	0.10
**Redundancy**	1.61e-01	1.72e-02	1.58e-01	2.03e-02	3.05	99	0.003	0.14	0.05	0.24

TABLE S1. Results for the statistical comparison between human structural connectivity networks, and corresponding geometry-preserving rewired nulls.

Contrast	Empirical Mean	Empirical SD	Null Mean	Null SD	tStat	df	pVal	Hedges g	95% CI Lower	95%CI Upper
**Synergy**	1.84e-01	1.94e-02	1.33e-01	2.02e-02	33.89	99	$p < 0.001$	2.56	2.29	2.91
**Unique distant**	4.97e-01	2.15e-02	2.60e-01	7.27e-03	111.42	99	$p < 0.001$	14.68	13.03	17.14
**Unique proximal**	1.58e-01	1.10e-02	2.50e-01	8.11e-03	-59.16	99	$p < 0.001$	-9.46	-10.65	-8.57
**Redundancy**	1.61e-01	1.72e-02	3.56e-01	2.99e-02	-116.18	99	$p < 0.001$	-7.98	-9.08	-7.20

TABLE S2. Results for the statistical comparison between human structural connectivity networks, and corresponding degree-preserving rewired nulls.

Contrast	Empirical Mean	Empirical SD	Null Mean	Null SD	tStat	df	pVal	Hedges g	95% CI Lower	95%CI Upper
**Synergy**	2.29e-01	3.45e-02	1.64e-01	2.31e-02	22.93	99	$p < 0.001$	2.22	1.97	2.57
**Unique distant**	2.71e-01	4.95e-02	3.71e-01	3.94e-02	-33.07	99	$p < 0.001$	-2.21	-2.65	-1.91
**Unique proximal**	2.67e-01	3.40e-02	1.91e-01	1.39e-02	24.56	99	$p < 0.001$	2.92	2.59	3.38
**Redundancy**	1.83e-01	3.74e-02	2.31e-01	3.47e-02	-20.42	99	$p < 0.001$	-1.33	-1.57	-1.15

TABLE S3. Results for the statistical comparison between human functional connectivity networks, and corresponding geometry-preserving rewired nulls.

Contrast	Empirical FC Mean	FC SD SD	SC Mean	SC SD	tStat	df	pVal	Hedges g	95% CI Lower	95%CI Upper
**Synergy**	2.29e-01	3.45e-02	1.84e-01	1.94e-02	14.23	99	$p < 0.001$	1.62	1.40	1.91
**Unique distant**	2.71e-01	4.95e-02	4.97e-01	2.15e-02	-39.13	99	$p < 0.001$	-5.88	-6.85	-5.23
**Unique proximal**	2.67e-01	3.40e-02	1.58e-01	1.10e-02	29.96	99	$p < 0.001$	4.27	3.80	4.94
**Redundancy**	1.83e-01	3.74e-02	1.61e-01	1.72e-02	6.94	99	$p < 0.001$	0.75	0.52	1.04

TABLE S4. Results for the statistical comparison between human structural connectivity networks, and corresponding functional connectivity networks, thresholded to have the same network density.

Contrast	Empirical Mean	Empirical SD	Null Mean	Null SD	tStat	df	pVal	Hedges g	95% CI Lower	95%CI Upper
**Synergy**	9.19e-02	4.46e-02	6.07e-02	4.22e-02	48.52	219	$p < 0.001$	0.72	0.61	0.88
**Unique distant**	3.43e-01	3.38e-02	3.61e-01	4.24e-02	-9.66	219	$p < 0.001$	-0.45	-0.55	-0.35
**Unique proximal**	2.19e-01	2.31e-02	1.81e-01	1.54e-02	31.22	219	$p < 0.001$	1.96	1.77	2.20
**Redundancy**	3.45e-01	4.94e-02	3.98e-01	5.93e-02	-32.95	219	$p < 0.001$	-0.96	-1.12	-0.83

TABLE S5. Results for the statistical comparison between mammalian structural connectivity networks, and corresponding geometry-preserving rewired nulls.

References

[1] Paul L Williams and Randall D Beer. Nonnegative decomposition of multivariate information. *arXiv:1004.2515*, 2010.
[2] Daniel Chicharro and Stefano Panzeri. Synergy and redundancy in dual decompositions of mutual information gain and information loss. *Entropy*, 19(2):71, 2017.
[3] Jason Crampton and George Loizou. Two partial orders on the set of antichains. *Research note*, 2000.
